# IL-33 Regulates the Phenotypic Transformation of Reactive Astrocytes via PENK-ERK/MAPK Pathway in Parkinson’s Disease

**DOI:** 10.1007/s12264-025-01566-2

**Published:** 2026-01-03

**Authors:** Yi Qu, Zhijuan Mao, Danlei Wang, Ke An, Haoheng Yu, Qixiong Qin, Jingyi Li, Yongjie Xiong, Zhe Min, Zheng Xue

**Affiliations:** 1 Department of Neurology, Tongji Hospital, Tongji Medical College, Huazhong University of Science and Technology, Wuhan, 430030 China; 2 Shandong Key Laboratory of Neurorehabilitation, School of Life Sciences and Health, University of Health and Rehabilitation Sciences, Qingdao, 266071 China; 3 Department of Neurology, The Second Affiliated Hospital of Guangxi Medical University, Nanning, 530021 China

**Keywords:** Parkinson’s disease, Neuroinflammation, Astrocytes, Interleukin-33, Preproenkephalin

## Abstract

Interleukin-33 (IL-33) regulates immune responses in central nervous system diseases. This study investigates the effect of IL-33 on astrocyte phenotypic transformation in Parkinson’s disease (PD). The associations of IL-33, soluble growth-stimulating expression gene 2 (sST2), with PD severity and clinical symptoms were examined. IL-33 supplementation and knockdown were applied *in vivo* and *in vitro* to assess IL-33’s impact on neuron loss, astrocyte polarization, and inflammation. Transcriptome sequencing was conducted to identify hub genes and pathways regulated by IL-33 in astrocytes, with validated in primary astrocytes. Plasma sST2 levels were elevated in PD patients and correlated with PD severity, while IL-33 decreased with disease progression. In PD models, IL-33 supplementation improved PD-like symptoms and A2 astrocyte polarization. Conversely, IL-33 knockdown worsened PD-like symptoms and neurotoxic polarization. RNA-seq identified the PENK-ERK/MAPK pathway as the key regulator of IL-33-mediated astrocyte transformation. In conclusion, IL-33 plays a crucial role in regulating astrocytes in PD.

**Supplementary Information:**

The online version contains supplementary material available at 10.1007/s12264-025-01566-2.

## Introduction

Parkinson’s disease (PD) is the second most prevalent neurodegenerative disorder, characterized by dopaminergic (DA) neuronal loss in the nigrostriatal pathway [1]. Clinically, PD presents with motor symptoms such as resting tremor, bradykinesia, rigidity, and postural instability, alongside non-motor symptoms including cognitive decline and sleep disturbances [2]. The chronic and progressive nature of PD imposes significant caregiving and economic burdens on both families and society [3].

Neuroinflammation plays a pivotal role in PD pathogenesis, primarily through the activation of glial cells [4]. Chronic activation of glia leads to the release of pro-inflammatory cytokines, which can induce neuronal toxicity and contribute to neurodegeneration in the central nervous system (CNS) [5]. Astrocytes, a type of glial cell, have been identified as having multiple roles in PD progression [6, 7], exhibiting both neurotoxic and neuroprotective effects [8]. Therefore, understanding the mechanisms by which astrocytes modulate inflammation is crucial for developing therapeutic strategies for PD.

Interleukin 33 (IL-33), a cytokine belonging to the IL-1 family, activates T helper 2 (Th2) cells by binding to the growth-stimulating expression gene 2 (ST2) receptor, thereby inducing inflammatory responses [9]. Notably, IL-33 and its receptors are highly expressed in the CNS, where they influence various pathological processes [10]. Studies have demonstrated that IL-33 released from astrocytes enhances microglial clearance of synapses and aids in neural circuit remodeling during CNS development [11]. Conversely, IL-33 released from neurons promotes microglial phagocytosis and regulates memory formation [12]. Despite its potential significance, IL-33 has been underexplored in the context of PD. Existing research indicates that IL-33 expression is elevated in the midbrain and striatum of PD patients compared to controls [13]. Activated mast cells, which are targeted by IL-33 in the brain, may trigger neuroinflammation, thereby contributing to PD progression [13]. *In vitro* studies also demonstrate that astrocytes induced by 1-Methyl-4-phenyl-pyridinium (MPP^+^) exhibit increased IL-33 levels [14]. Plasma levels of soluble ST2 (sST2) are higher in PD patients than in healthy controls (HC), correlating with cognitive decline, particularly affecting working memory, executive function, and visuospatial abilities [15]. Additionally, serum IL-33 levels are significantly elevated in PD patients [16], suggesting involvement of the IL-33/ST2 signaling pathway in PD progression.

This study analyzed plasma IL-33 and sST2 levels with disease severity and clinical phenotypes in PD patients from Central China. On the other hand, we established acute PD mouse models via 1-methyl-4-phenyl-1,2,3,6-tetrahydropyridine (MPTP) intraperitoneal injection and a primary astrocyte model stimulated by MPP^+^ to elucidate the role of IL-33 in astrocyte activation and PD pathogenesis. By achieving these objectives, the study seeks to elucidate the role and therapeutic potential of IL-33 in PD, potentially offering new avenues for treatment.

## Material and Methods

### Participants

The present study enrolled 180 patients who visited Tongji Hospital, Tongji Medical College, Huazhong University of Science and Technology between November 2021 and October 2022. Additionally, 80 HC subjects were recruited from local communities and medical centers. PD participants were diagnosed according to the 2015 Movement Disorder Society (MDS) clinical diagnostic criteria [17]. Exclusion criteria included the diagnosis of Parkinsonism-plus syndromes, history of specific surgeries such as stereotactic nerve nuclei lesions and deep brain stimulation, or significant medical history (e.g., psychiatric symptoms, cancer, serious cardiovascular complications). Following these criteria, 74 subjects were excluded, resulting in a final cohort of 186 participants (Fig. S1). Informed consent was obtained from all participants, and the study received ethical approval from the Ethics Committee of Tongji Hospital (TJ-IRB20220752).

### Clinical Assessments

Basic demographic and clinical information were collected, and disease severity was evaluated in PD patients. Motor function assessments were conducted during the "off" phase (6–8 h after the last medication dose), while other evaluations occurred in the "on" phase (1 h post-medication). Medication use within one week was calculated for each patient in terms of the levodopa equivalent dose (LEDD) [18]. The midbrain area was imaged using transcranial ultrasound (TCS), performed and reviewed by specialized physicians [19].

### Plasma IL-33 and sST2 Measurement

EDTA-anticoagulated blood samples were centrifuged at 1,800 *g* for 20 min within 2 h of collection. The plasma was aliquoted and stored at –80℃ until use. Plasma IL-33 and sST2 levels were measured using Enzyme-Linked Immunosorbent Assay (ELISA) kits (Human IL-33 ELISA Kit, RK04877, Abclonal, Wuhan, China; Human ST2 ELISA Kit, KE00293, Proteintech, Wuhan, China), following the manufacturer’s protocol. The inter- and intra-assay coefficients of variation, calculated from duplicate measurements, were less than 15%.

### Animals

The study utilized male C57BL/6J mice, aged 7 weeks and weighing between 24 and 27 g, purchased from Gempharmatech (Nanjing, China). Mice were housed under specific pathogen-free (SPF) conditions at the Tongji Hospital Animal Center, with a 12 h light/dark cycle (23 ± 2℃) and ad libitum access to food and water. The experimental procedures received approval from the Tongji Hospital Animal Ethics Committee (282-20231006).

### Model Establishment and Grouping

#### MPTP-Induced Acute PD Model

Forty mice were randomly assigned to four groups: Control (*n =* 10), MPTP-1 day (*n =* 10), MPTP-3 day (*n =* 10), and MPTP-7 day (*n =* 10). The MPTP groups received four intraperitoneal injections of 20 mg/kg MPTP (100 mg MPTP dissolved in 20 mL PBS; S4732, Selleck, Shanghai, China) at 2-h intervals within a single day. The control group received an equivalent volume of phosphate-buffered saline (PBS) intraperitoneally. Subsequent procedures were conducted at 1, 3, and 7 days post-initial injection.

#### Substantia Nigra (SN) rIL-33 Stereotactic Injection Model

Forty mice were randomly assigned to four groups: PBS control (*n =* 10), recombinant IL-33 (rIL-33; *n =* 10), MPTP (*n =* 10), and MPTP+rIL-33 (*n =* 10). First, the MPTP model was established, referring to 2.5. On day 3 post-MPTP administration, 100 ng rIL-33 (50 μg IL-33 dissolved in 500 μL of distilled water; HYP7218, MCE, Shanghai, China) was stereotaxically injected into the SN of the rIL-33 and MPTP+rIL-33 groups. The injection coordinates were: anteroposterior (AP) −3.0 mm, mediolateral (ML) +1.2 mm, dorsoventral (DV) −4.7 mm, with bregma as the reference point. Other groups received equivalent PBS injections. Evaluations were performed after 7 days post-MPTP injection.

#### SN Astrocyte-Specific Il33 Knockdown Model

Forty mice were randomized into two groups: AAV-Control (*n =* 20) and AAV-hGFAP-sh*Il33* (*n =* 20). Astrocyte-specific *Il33* gene knockdown was achieved using an adeno-associated virus (AAV) vector carrying a human glial fibrillary acidic protein (GFAP) promoter driving shRNA targeting murine *Il33* (Tsingke, Beijing, China). Mice received bilateral SN injections of either AAV-hGFAP-sh*Il33* (2.07×10^13^ vgs/mL, 1.5 μL/side) or AAV-CMV-EGFP (1.01×10^13^ vgs/mL, 1.5 μL/side) using the following coordinates: AP −3.0 mm, ML ±1.2 mm, DV −4.7 mm. Four weeks post-AAV injection, successful knockdown in astrocytes was confirmed by immunofluorescence (IF) for IL-33 and GFAP. Mice were then randomly divided into PBS (*n =* 10/group) and MPTP (*n =* 10/group) subgroups, with models established referring to 2.5. Evaluations were conducted at 7 days post-MPTP injection.

### Primary Astrocytes Culture and Treatment

Primary cultures of murine astrocytes were derived from C57BL/6J mouse pups aged 0–3 days. Briefly, the brain samples were dissociated into single cell suspensions and cultured in Dulbecco’s modified Eagle’s medium (DMEM)/F12 with 10% fetal bovine serum (FBS) at 37℃ and 5% CO_2_. Medium changes were performed every 72 h for 12 days. Once the primary astrocytes reached full confluence, flasks were shaken at 180 rpm for about 18 h at 37°C, followed by vigorous tapping to detach microglia and oligodendrocyte progenitor cells. Cells were then subcultured every 3–5 days. Astrocytes with 95% purity, confirmed by IF, were deemed suitable for further experiments.

### Astrocytes Treatment and Transfection

To set up a PD model *in vitro*, astrocytes were treated with MPP^+^ (20mmol/L, D048, Sigma-Aldrich, St. Louis, MO, USA). MPP^+^ treatment occurred for 0, 12, 24, or 48 h. Following the treatments, cells were washed with PBS and incubated with fresh medium for another 24 h before collecting conditioned supernatants. Supernatants were collected and processed immediately by centrifugation at 15,000 × *g* for 15 min at 4°C for subsequent ELISA detection.

siRNA targeting *Il33*, or *Il33* plasmid (Tsingke), was transfected into astrocytes using Lipofectamine™ 3000 (Invitrogen, USA) in Opti-MEM reduced serum medium (Gibco, NYC, USA) according to the manufacturer's instructions. After 24 h, the transfected cells were exposed to either MPP^+^ or PBS for 48 h before being collected for further experiments. For ELISA analysis, cells were incubated for an additional 24 h to harvest the supernatants.

To investigate whether the ERK/MAPK pathway is involved, a specific MEK inhibitor (U0126, MCE) was employed. U0126 was dissolved in DMSO to prepare a 1 mmol/L stock solution for long-term storage. For astrocyte treatment, a working concentration of 10 μmol/L U0126 was selected, as it has been previously validated to effectively inhibit the phosphorylation of ERK1/2[20]. Astrocytes were first preincubated with U0126 for 8 h, and were exposed to PBS or MPP^+^ for 48 h. Cells were collected for subsequent analyses.

### IF Staining and Analyses

Mice were anesthetized and subjected to transcardial perfusion with 4% paraformaldehyde (PFA). The whole brain was stabilized in PFA for 24 hours and subsequently dehydrated in a PBS solution containing 30% sucrose until the samples sank to the bottom. Coronal sections (10 μm thick) of the SN were cut using a freezing microtome (Thermo Fisher Scientific, Waltham, MA, USA). Frozen brain slices were fixed, blocked, and incubated with primary antibodies overnight at 4℃ (Table S1). Afterward, the slices were incubated with secondary antibodies at room temperature in the dark for 1 h, and were then mounted with an anti-fade mounting containing DAPI (Beyotime, Shanghai, China). Images were acquired using a confocal microscope (FV1200, Olympus, Japan). The number of TH^+^ DA neurons was determined by counting the TH^+^ cells and standardized by the average number of TH^+^ cells in the control group. The percentage of C3/S100A10^+^GFAP^+^ double-positive cell area was calculated by calculating the area of C3^+^GFAP^+^ or S100A10^+^GFAP^+^ with the area of the GFAP^+^ ratio from at least 4 representative images per mouse.

### Quantitative Real-time PCR (qPCR) Analysis

Total RNA from mouse SN tissues and cells was extracted using TRIzol reagent (AGBio, Changsha, China) and quantified with a NanoDrop spectrophotometer (IMPLEN, Munich, Germany). cDNA synthesis was performed using Hifair® III 1st Strand cDNA Synthesis SuperMix (Yeasen, Shanghai, China) following the manufacturer's instructions. qPCR was carried out using Hieff® qPCR SYBR® Green Master Mix to evaluate gene expression with the CFX Connect Detection System (Bio-Rad, Hercules, CA, USA). Primer sequences are provided in Table S2. Relative mRNA expression levels were analyzed by the 2^−ΔΔCt^ method, with *Actb* as the reference gene.

### Western Blot (WB) Analysis

Total protein from SN tissues and cells was extracted using RIPA buffer (Sevicebio, Wuhan, China) containing a protease inhibitor cocktail and phenylmethylsulfonyl fluoride (PMSF). Cytoplasmic and nuclear proteins of astrocytes were isolated using the cytoplasmic and nuclear protein extraction kit (Beyotime), following the manufacturer's protocol. Equal amounts of protein were loaded onto an SDS-polyacrylamide gel for electrophoresis and then transferred to nitrocellulose membranes. These membranes were blocked with 5% non-fat skim milk and incubated with primary antibodies at 4℃ overnight (Table S3). Secondary antibody incubations were performed for 1 h at room temperature. WB protein bands were visualized by enhanced chemiluminescence, and images were captured with the GelView 6000 Pro imaging system (Biolight, Guangzhou, China).

### ELISA

The levels of IL-33 (KE10054, Proteintech), sST2 (JM-12884M1, Jingmei, Ningbo, China), and IL-1β (KE10003, Proteintech) in the SN and astrocyte supernatants were determined by ELISA kits, following the manufacturer’s protocols.

### High-throughput Transcriptome Sequencing (RNA-seq)

RNA-seq was performed using primary astrocytes transfected with si*Il33* and stimulated with MPP^+^. Total RNA was extracted, flash-frozen in liquid nitrogen, and shipped on dry ice for analysis (Novogene, Beijing, China). Quality control was performed by the Agilent Bioanalyzer 2100, ensuring an RNA integrity number (RIN) of ≥ 7.0. Library preparation was performed using the TruSeq Stranded Total RNA Kit (Illumina), and sequencing was conducted on the NovaSeq 6000 platform (150 bp paired-end reads, ≥ 30 million reads per sample). Differential gene expression (DEGs) analysis was conducted using the ‘limma’ package, with the following criteria for identifying DEGs: |Log_2_fold change (FC)| > 1.0 and adjusted *P*-value < 0.01.

### Statistical Analysis

Statistical analyses were conducted using R software (version 4.1.3) and GraphPad Prism (version 9). In the population study, participant characteristics were compared between groups using the Kruskal-Wallis test for continuous variables and the Chi-square test for categorical variables. Data were normalized using the ‘car’ package if they did not meet normal distribution. Multiple linear regression models were applied to explore correlations of plasma IL-33 and sST2 levels with PD features. For both *in vivo* and *in vitro* analyses, group comparisons were performed using a two-tailed Student’s *t* test for 2 groups and two-way analysis of variance (ANOVA) for multiple groups. *P*-values < 0.05 were considered statistically significant.

## Results

### Plasma IL-33 and sST2 Levels Among Movement Disorder Populations

**Fig. 1:**
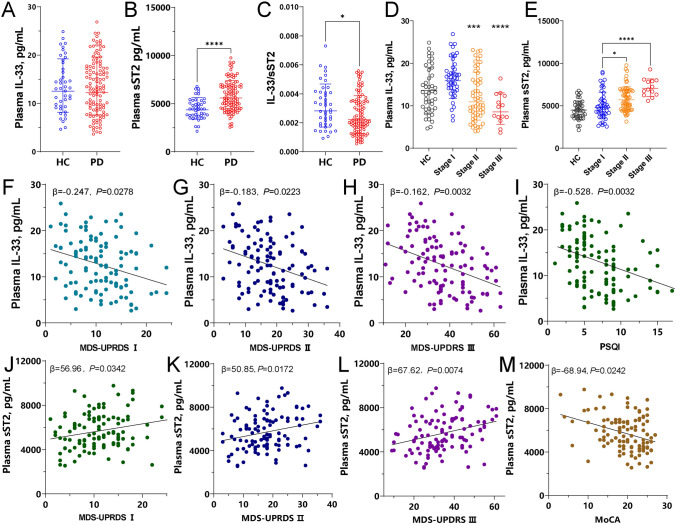
Associations of plasma IL-33 and sST2 levels with PD symptoms. **A** No significant differences were found of plasma IL-33 among groups; **B** Plasma sST2 levels were significantly increased in patients with PD compared to HC (*P* = 0.0010) and ET (*P* = 0.0104); **C** IL-33/ST2 ratio was significant decreased in PD patients (*P* = 0.0265); **D** Plasma Il-33 levels gradually decreased as H&Y stages progressed (*P* < 0.0001); **E** Plasma sST2 levels gradually elevated as H&Y stages progressed (*P* = 0.0071); **F–I** Lower levels of IL-33 were associated with worsen symptoms in PD patients, including MDS-UPDRS part I (*P* = 0.0278;** E**, part II (*P* = 0.0223; F), part III (*P* = 0.0032; G) and PSQI (0.0032; H); **J–M** Higher levels of sST2 were associated with worsen symptoms in PD patients, including MDS-UPDRS part I (*P* = 0.0342; I), part II (*P* = 0.0172; **J**), part III (*P* = 0.0061; **K**) and MoCA (*P* = 0.0242; **L**); Data are presented as mean ± SD.

This study enrolled 155 subjects aged 40-85 years, including 112 PD and 43 patients. Demographic details are presented in Table S4. No significant differences in IL-33 levels were observed between PD and HC groups (Fig. 1A). However, differences were detected in plasma sST2 (*P* < 0.0001, Fig. 1B) and IL-33/ST2 ratio (*P* = 0.02655, Fig. 1C).

ROC curve analysis was performed to evaluate the diagnostic and discriminative utility of plasma sST2 in PD (Table S5). It demonstrated moderate to high discriminatory power in distinguishing PD from HC (AUC = 0.702; Fig. S2A), ET (AUC = 0.708; Fig. S2C), and dystonia (AUC = 0.842; Fig. S2D), but not from PSP or MSA (AUC = 0.548; Fig. S2B). The cut-off value for plasma sST2 to differentiate PD from HC was 5,441 pg/mL, yielding a sensitivity of 51.8% and specificity of 79.1% at this threshold.

### Associations of Plasma IL-33 and sST2 Levels with Disease Severity in PD Patients

Subgroup analyses were performed to examine the correlations of IL-33 and sST2 with disease severity and clinical assessment indices in PD patients. The cohort included 112 PD patients with H&Y stages 1–3, stratified into stage I (1–1.5), II (2–2.5), III (3) groups based on disease severity (Table S3), containing 43, 55, and 14 patients, respectively. Plasma IL-33 levels gradually decreased with disease progression (*P* < 0.0001, Fig. 1D), while sST2 concentrations gradually increased with disease severity (*P* = 0.0071; Fig. 1E).

**Fig. 2:**
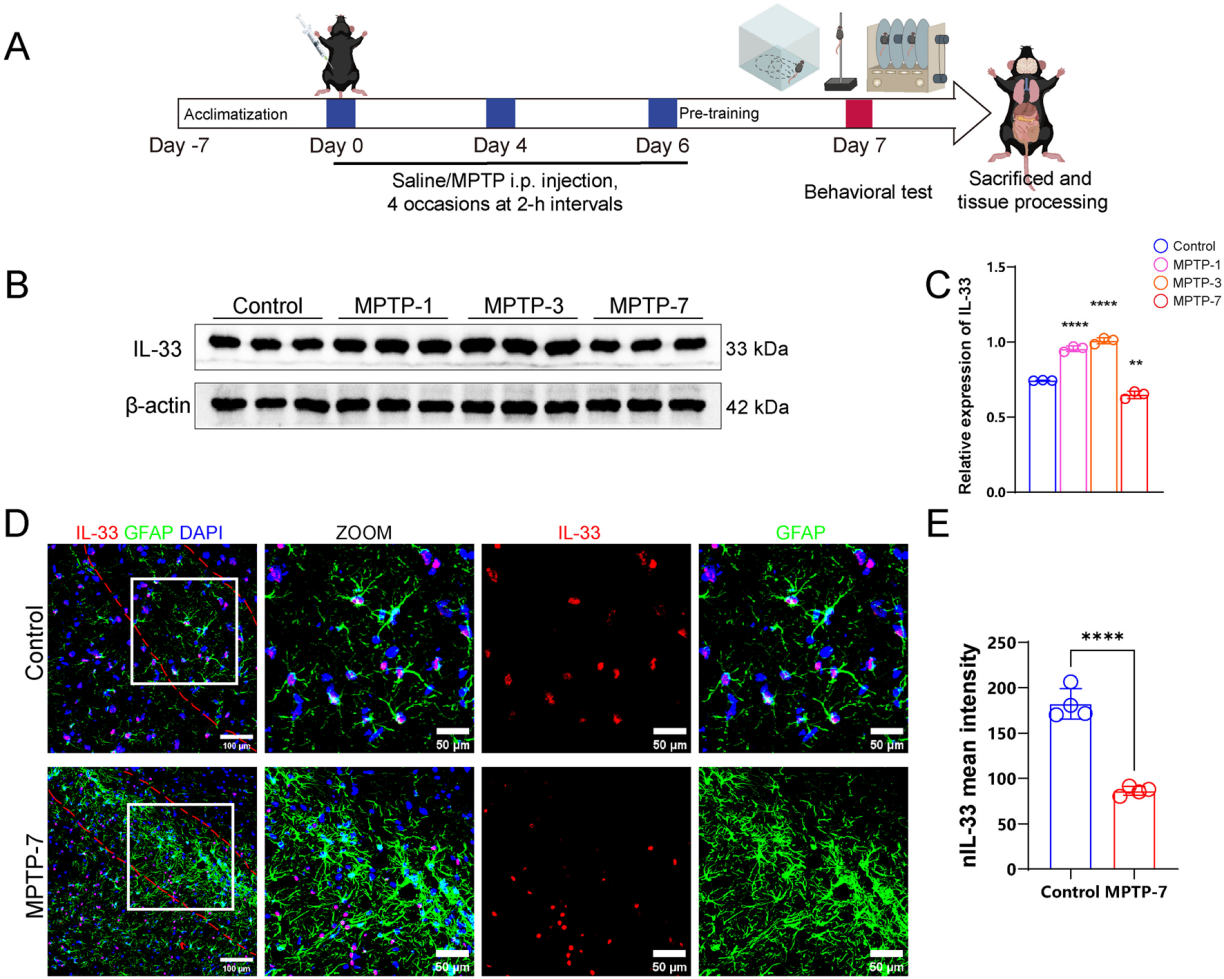
Expression and co-localization of IL-33 in the SN. **A** Flowchart illustrating the construction of MPTP mice model;** B**, **C** Protein expression of IL-33 in the SN assessed by WB (**D**) and quantitative analyses (**E**) among groups, *n =* 3 per group; **D** Representative IF images of IL-33 with GFAP in the SN, scale bar = 25 μm; **E** Quantification of IL-33 average intensity in control and MPTP-7 mice, *n =* 4 per group; Data are presented as mean ± SD. Significances were assessed using two-way ANOVA, *P < 0.05, **P < 0.01, ***P < 0.001, ****P < 0.0001.

Multivariate linear regression analyses were performed to examine the associations between IL-33 or sST2 levels and clinical parameters (Table S6). Lower IL-33 levels were associated with worse MDS-UPDRS scores in Part I (*P* = 0.0278; Fig. 1F), Part II (*P* = 0.0223; Fig. 1G), and Part III (*P* = 0.0032; Fig. 1H). Additionally, they were negatively correlated with PSQI scores (*P* = 0.0032; Fig. 1I). On the other hand, plasma sST2 showed a noticeable association with PD symptoms. As PD motor symptoms worsened, plasma sST2 levels gradually increased. Specifically, MDS-UPDRS part I (*P* = 0.0342; Fig. 1J) and part III (*P* = 0.0061; Fig. 1L) scores were positively related to sST2. Furthermore, elevated sST2 levels were associated with LEDD intake (*P* = 0.0248; Fig. S2F) and reduced TCS midbrain area (*P* = 0.0039; Fig. S2G). Non-motor symptoms in PD also showed noteworthy relationships with plasma sST2 levels. MDS-UPDRS Part II scores, which assess non-motor symptoms, positively correlated with sST2 concentrations (*P* = 0.0172; Fig. 2K). In particular, cognitive impairment, evaluated by MMSE (*P* = 0.0114; Fig. S2G) and MoCA (*P* = 0.0242; Fig. 2M), was inversely associated with sST2.

Plasma sST2 levels showed significant correlations with inflammation markers. For instance, sST2 levels positively correlated with neutrophil counts (*P* = 0.0008; Fig. S2H) and NLR (*P* = 0.0078; Fig. S2K). hs-CRP concentrations also positively correlated with sST2 (*P* = 0.0234; Fig. S2J). These findings suggest that sST2 serves as a biomarker of inflammatory activation in PD, with higher levels reflecting disease-associated inflammation. Besides, increased sST2 was closely associated with reduced transferrin levels (*P* = 0.0148; Fig. S2K), possibly reflecting increased brain iron deposition in PD.

### Expression and Co-localization of IL-33 in Acute MPTP Mouse Model

An acute MPTP-induced PD mouse model was established, with experimental procedures shown in Fig. 2A. To assess the expression of IL-33 in the SN, qPCR and WB were performed. qPCR results demonstrated a progressive upregulation of the *Il33* gene in MPTP mice at first, reaching peak levels at day 3 before declining to significantly lower levels than controls by day 7 (Fig. S3A). The expression of *Il1rl1*, the gene encoding ST2, was upregulated in MPTP mice, peaking on day 3 and remaining elevated above control levels at day 7 (Fig. S3B). WB results were consistent with qPCR findings, which showed clear increases in IL-33 protein levels at day 3, followed by a marked decrease by day 7 (Fig. 2B, C). Additionally, ST2 levels were most prominent on day 3 and, although showing no significant difference from controls at day 7, a slight upward trend was observed (Fig. S3C, D).

Subcellular localization of IL-33 and ST2 in the SN was examined. IF staining demonstrated that both IL-33 and ST2 were primarily co-expressed in the same cells of SN. Notably, IL-33 was localized specifically in the nucleus, while ST2 was found on the cell membrane (Fig. S3E). To further delineate IL-33's cellular localization, co-IF staining was performed using neuron l marker neuronal nuclei (NeuN), DA neuron marker tyrosine kinase (TH), microglial marker ionized calcium-binding adapter molecule 1 (iba1), astrocyte marker GFAP, and oligodendrocyte marker oligodendrocyte lineage transcription factor 2 (OLIG2) in the SN (Fig. S3F). The results revealed that IL-33 predominantly co-localized with GFAP and OLIG2, with minimal or no co-localization with NeuN, TH, or iba1, indicating that IL-33 is mainly expressed in astrocytes and oligodendrocytes. Similarly, IF staining of ST2 with TH, iba1, GFAP, and OLIG2 showed that ST2 was also primarily expressed in astrocytes and oligodendrocytes (Fig. S3G). Notably, while we did not detect ST2 co-localization with microglia, prior studies have reported ST2 expression in microglia [21]. We hypothesize this discrepancy may stem from two key, context-dependent factors. On one hand, the proportion of ST2^+^ microglia in the SN could be extremely low, failing to reach the detection threshold. On the other hand, microglia specifically within the SN may inherently lack ST2 expression. This reflects a region-specific expression pattern of ST2, which differs from other brain regions.

**Fig. 3:**
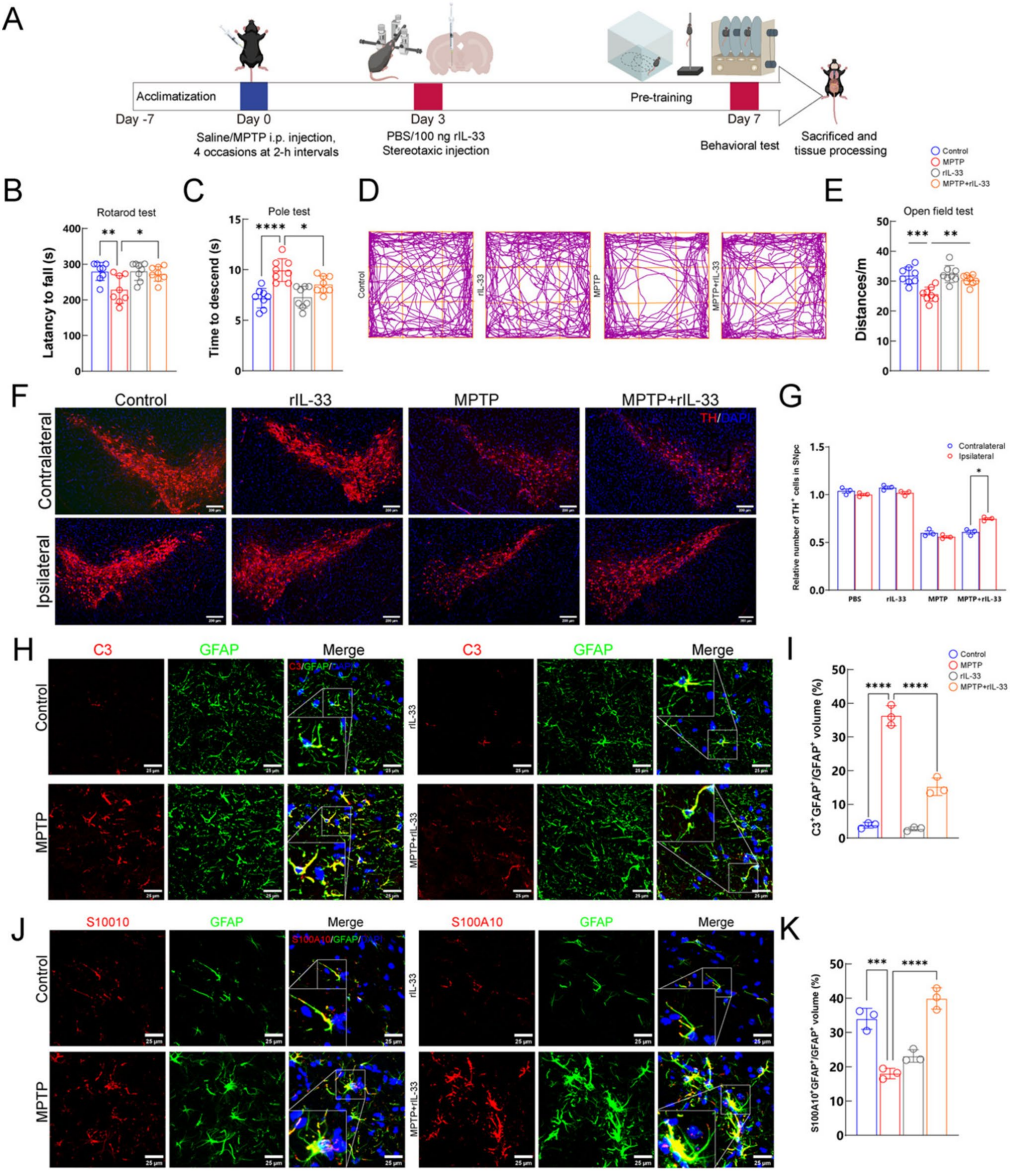
rIL-33 supplementation ameliorates PD-like symptoms and neuro-inflammation in the MPTP mouse model. **A** Flowchart illustrating the construction of rIL-33 SN-injected MPTP mouse model; **B**, **C** Quantitative analyses of latency to fall in the rotarod test (B) and time to descend in the pole test (**C**), *n =* 8 per group; **D** OFT showing representative images of mouse movement trajectories; **E** Quantitative analyses of total distance in OFT, *n =* 8 per group; **F, G** Representative IF images of TH in the bilateral SN (**F**) and quantification of TH^+^ cells (**G**), scale bar = 200 μm, *n =* 3 per group; **H** Representative confocal images of C3 (red) and GFAP (green) in the SN, scale bar = 25 μm; **I** Quantitative analysis of the ratio of C3^+^GFAP^+^ to GFAP^+^ area in the SN, *n =* 3 per group; **J** Representative confocal images of S100A10 (red) and GFAP (green) in the SN, scale bar = 25 μm; **K** Quantitative analysis of the ratio of S100A10^+^GFAP^+^ to GFAP^+^ area in the SN, *n =* 3 per group; Significances were assessed using two-way ANOVA. *P < 0.05, **P < 0.01, ***P < 0.001, ****P < 0.0001.

Given the pivotal role of astrocytes in mediating neuroinflammatory responses, this study specifically focused on characterizing IL-33 expression and its functional roles in astrocytes within the SN. WB analysis revealed that protein levels of GFAP were gradually increased in MPTP mice (Fig. S3H, I), accompanied by elevation of C3 and S100A10 (Fig. S4H, J, K), indicating significant activation of astrocytes with both neurotoxic and protective roles in the MPTP model. A significant increase in the number of GFAP^+^ cells and the intensity of GFAP fluorescence was observed in MPTP mice (Fig. 3D). Subcellular analyses revealed reduced nuclear IL-33 (nIL-33) intensity of SN in MPTP-7d mice (Fig. 2D, E).

### rIL-33 Supplementation Improves PD-like Symptoms and Neuroinflammation in MPTP Mouse Model

A significant IL-33 level decrease in SN of MPTP mice by day 7 was found (Fig. 2B, D). Therefore, we administered recombinant IL-33 (rIL-33) via stereotaxic injection into the SN, with experimental procedures outlined in Fig. 3A. WB confirmed the successful delivery of rIL-33, with elevated IL-33 levels in the SN of treated mice compared to controls (Fig. S4A, B). Motor functions were evaluated using the rotarod, pole, and open field tests. MPTP mice exhibited marked reduction in rotarod latency (Fig. 3B), prolonged pole descent time (Fig. 3C), along with a decrease in total distance traveled (Fig. 3D, E), all indicative of significant motor dysfunction. However, rIL-33-treated PD mice showed substantial improvements in these parameters, reflecting the amelioration of MPTP-induced motor deficits. The TH expression was used to assess damage to DA neurons in the SN. No significant loss of TH^+^ neurons was observed in rIL-33-treated mice compared to controls, confirming the safety of rIL-33 administration. Conversely, MPTP-induced mice exhibited bilateral loss of TH^+^ neurons, which was partially reversed by ipsilateral rIL-33 injection (Fig. 3F, G). WB further corroborated the IF findings, showing reduced TH protein in MPTP mice, which was rescued by rIL-33 treatment (Fig. S4C, D).

Next, we investigated the impact of rIL-33 on astrocyte polarization toward A1/A2 phenotypes in the SN. Comparable shapes were found between rIL-33-treated and control groups (Fig. 3H), confirming that rIL-33 alone does not induce astrocyte activation. WB results demonstrated that rIL-33 attenuated GFAP increase in MPTP mice (Fig. S4E, F). Co-IF staining of C3 with GFAP showed an evident growth in the ratio of C3^+^GFAP^+^ to GFAP^+^ area in MPTP mice, which was decreased after rIL-33 injection (Fig. 3H, I). In contrast, the ratio of S100A10^+^GFAP^+^ to GFAP^+^ area in MPTP mice was reduced, but it was increased after rIL-33 treatment (Fig. 3J, K). WB analysis revealed that the proteins of C3 and S100A10 were increased in the SN of MPTP mice (Fig. S4E–G). After rIL-33 supplementation, C3 levels significantly decreased, while S100A10 further increased.

To see how rIL-33 affects the neuroinflammatory environment in mice, microglial activation was checked. IF staining showed that microglia activation in the SN was similar between the rIL-33 group and PBS group, but MPTP-treated mice had much more activated microglia (Fig. S5A, C). In MPTP mice, the proportion of CD68^+^ and iba1^+^ double-positive microglia was much higher than in the control group. However, rIL-33 injection reduced this proportion, meaning it stopped pro-inflammatory microglia from being overactivated (Fig. S5A, B). MPTP mice also had more CD206^+^iba1^+^ microglia, and rIL-33 made this anti-inflammatory proportion even higher (Fig. S5C, D). Thus, rIL-33 can push microglia toward the anti-inflammatory type and reduce neuroinflammation.

These results suggest that SN injection of rIL-33 would suppress reactive astrocytes and promote neuroprotective polarization, which leads to the attenuation of DA neuron degeneration and motor deficits in the PD model.

### Astrocyte-specific *Il33* Knockdown Exacerbates PD-like Symptoms and Neuroinflammation in the MPTP Mouse Model

**Fig. 4:**
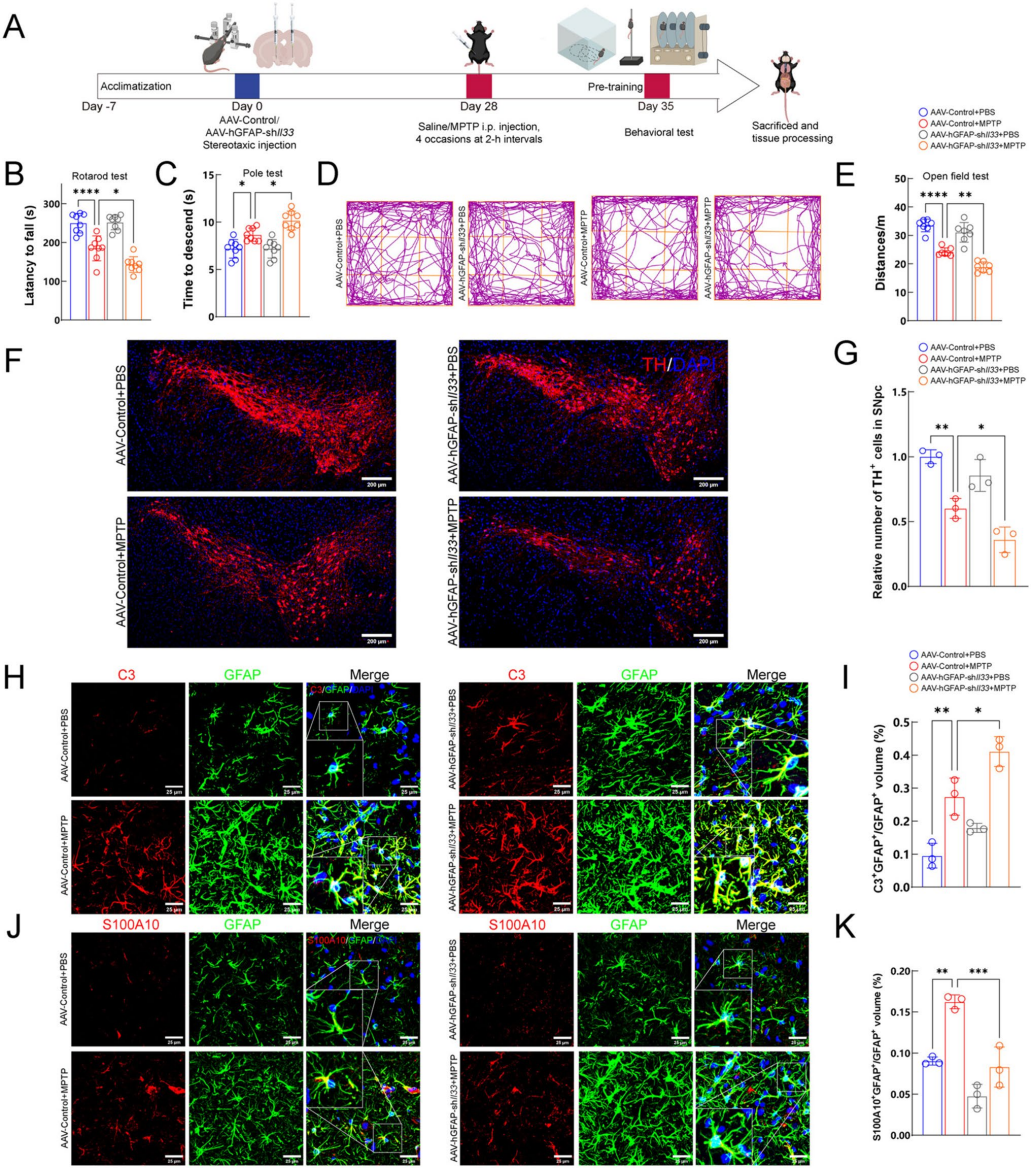
Astrocyte-specific *Il33* knockdown aggravates PD-like symptoms and neuroinflammation in the MPTP mouse model. **A** Flowchart illustrating the construction of AAV-hGFAP-sh*Il33* SN-injected MPTP mouse model; **B**, **C** Quantitative analyses of latency to fall in the rotarod test (**B**) and time to descend in the pole test (**C**), *n =* 8 per group; **D** OFT showing representative images of mouse movement trajectories; **E** Quantitative analyses of total distance in OFT, *n =* 8 per group; **F, G** Representative IF images of TH in the bilateral SN (**F**) and quantification of TH^+^ cells (**G**), scale bar = 200 μm, *n =* 3 per group; **H** Representative confocal images of C3 (red) and GFAP (green) in the SN, scale bar = 25 μm; **I** Quantitative analysis of the ratio of C3^+^GFAP^+^ to GFAP^+^ area in the SN, *n =* 3 per group; **J** Representative confocal images of S100A10 (red) and GFAP (green) in the SN, scale bar = 25 μm; **K** Quantitative analysis of the ratio of S100A10^+^GFAP^+^ to GFAP^+^ area in the SN, *n =* 3 per group; Significances were assessed using two-way ANOVA. *P < 0.05, **P < 0.01, ***P < 0.001, ****P < 0.0001.

An astrocyte-specific *Il33* knockdown model using AAV-mediated shRNA delivery targeting GFAP-positive cells was conducted (Fig. 4A). The knockdown efficiency was confirmed by IF at 28 days post-AAV administration, which revealed a significant reduction in the number of IL-33^+^GFAP^+^ cells in the SN of mice injected with AAV-hGFAP-sh*Il33* (Fig. S6A, B). Additionally, ELISA confirmed a decrease in IL-33 secreted levels in the SN (Fig. S6C), validating the successful establishment of the astrocyte-specific *Il33* knockdown model. For behavioral tests, MPTP mice injected with AAV-hGFAP-sh*Il33* exhibited a further decreased rotarod latency (Fig. 4B), increased pole climbing time (Fig. 4C), and a reduction in total distance (Fig. 4D, E) compared to AAV-Control mice. For DA neuron loss, the number of TH^+^ neurons of MPTP mice with *Il33* knockdown was significantly decreased compared to the AAV-Control MPTP group (Fig. 4F, G). TH protein levels of *Il33*-deficient mice were lower than those in normal MPTP mice (Fig. S6D, E).

Besides, an increase in the C3^+^/GFAP^+^ area in *Il33*-knockdown MPTP mice (Fig. 4H, I), while the S100A10^+^/GFAP^+^ area was reduced (Fig. 4J, K). WB analysis further supported these observations, with significantly higher C3 protein levels in *Il33*-knockdown MPTP mice (Fig. S6F, G) and lower S100A10 levels (Fig. S6F, H). Collectively, these data demonstrate that IL-33 deficiency promotes neurotoxic while inhibiting neuroprotective astrocytes, disrupting A1/A2 phenotypic balance. Moreover, the ratio of CD68^+^iba1^+^ area to iba1^+^ in the SN of MPTP mice was significantly elevated compared with the control group. This ratio was further increased following *Il33* knockdown (Fig. S7A, B). IL-1β levels in both the SN and serum were significantly elevated in MPTP mice (Fig. S6I, J), with a further increase observed in mice injected with AAV-hGFAP-sh*Il33*.

### Dynamic Changes of IL-33 and ST2 in MPP^+^-stimulated Astrocytes

**Fig. 5:**
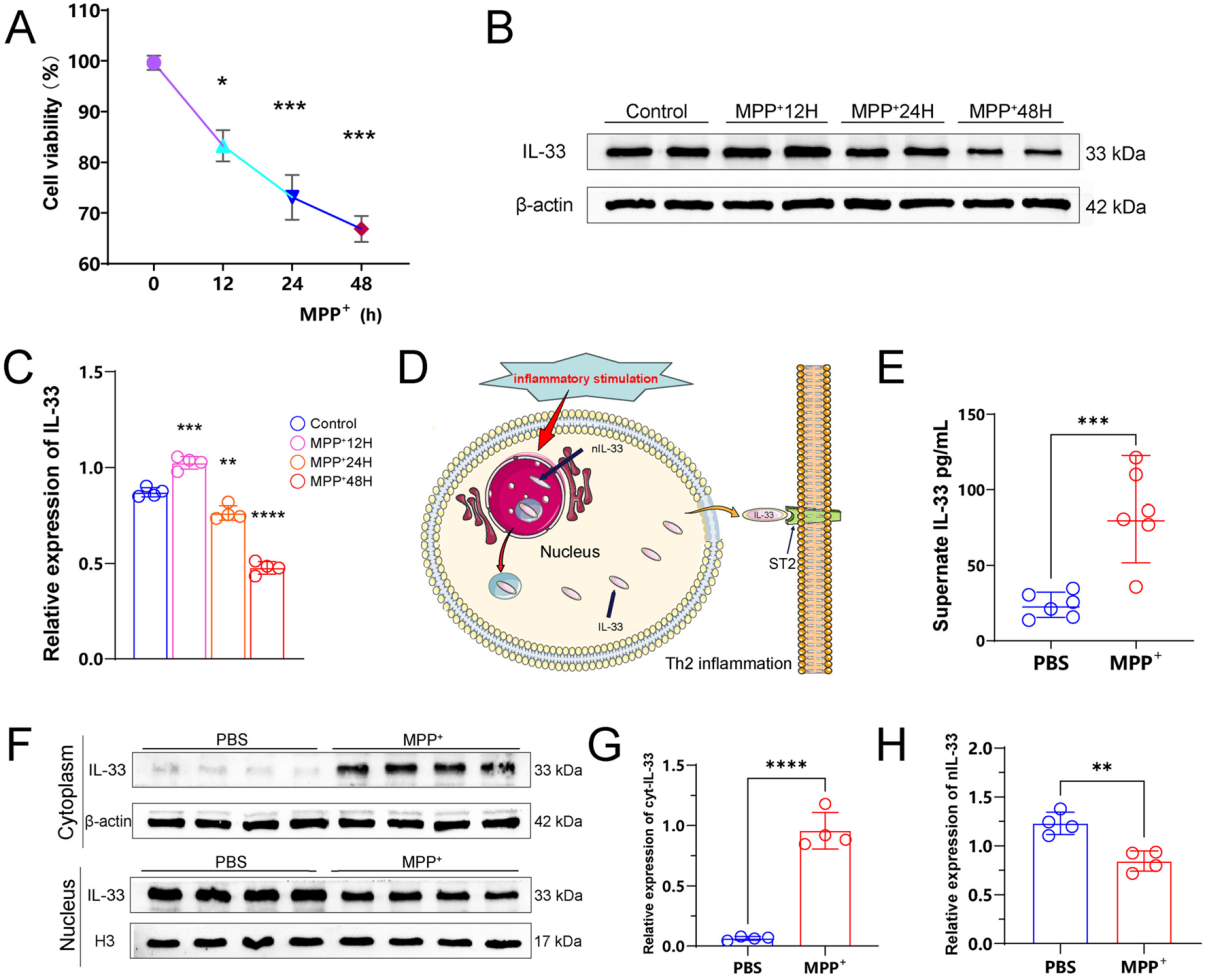
Dynamic expression of IL-33 in astrocytes. **A** Cell viability of MPP^+^-treated astrocytes in different time points assessed by CCK-8; **B, C** Protein expression of IL-33 in astrocytes assessed by WB (**B**) and quantitative analyses (**C**) among groups, *n =* 4 per group; **D** Schematic diagram illustrating IL-33 translocation from the nucleus to the cytoplasm; **E** Quantification of IL-33 levels in astrocyte-secreted supernatant detected by ELISA, *n =* 6 per group; **F–H** Protein expression of IL-33 in cytoplasm and nucleus assessed by WB (**F**), and quantitative analyses of the expression (**G**, **H**), *n =* 6 per group; Significances were assessed using two-way ANOVA. *P < 0.05, **P < 0.01, ***P < 0.001, ****P < 0.0001.

Primary astrocyte cultures were generated and characterized by IF staining, with purity ≥ 95% confirmed by GFAP^+^ cell quantification (Fig. S8A), and they were treated with MPP^+^ to establish an *in vitro* PD model. Astrocytes were exposed to 0.5 mmol/L MPP^+^ for 0, 12, 24, or 48 h, as illustrated in Fig. S8B. The viability of astrocytes gradually decreased with increasing concentrations of MPP^+^ from the CCK-8 test (Fig. S8C). Upon stimulation with 0.5 mmol/L MPP^+^, the viability of astrocytes was approximately 70%. Although the cells sustained substantial damage, they retained a certain level of viability, making this concentration suitable for subsequent experiments. In contrast, when the MPP^+^ concentration reached 1 mmol/L, astrocyte viability decreased sharply, resulting in the death of nearly half of the cells and rendering it unsuitable for subsequent experiments. Thus, 0.5 mmol/L MPP^+^ was selected for subsequent cell treatments. Additionally, MPP^+^ was applied to astrocytes at different time points (0, 12, 24, and 48 h). Results demonstrated that astrocyte viability gradually decreased with prolonged treatment duration (Fig. 5A).

After MPP^+^ stimulation, both A1 and A2 types are activated (Fig. S8D, E). However, the neuroprotective effect mediated by A2 astrocytes weakened with prolonged stimulation, leading to an imbalance between A1 and A2 astrocytes and further aggravating astrocyte damage. WB results showed a time-dependent elevation of C3, and a transient increase in S100A10 at 24 h (Fig. S8F–H). *Il33* mRNA levels were significantly upregulated after 12 h of MPP^+^ stimulation, then gradually decreased over time (Fig. S8I). At 48 h, *Il33* was evident lower than that in Controls. Protein levels of IL-33 were higher in astrocytes treated with MPP^+^ for 12 h than in Controls, but gradually decreased, with a significant downregulation observed at 48 h (Fig. 5B, C).

### IL-33 Translocated from Nucleus to Cytoplasm in Astrocytes Induced by MPP^+^

Accumulating evidence suggests that stimulation leads to the depletion of nuclear IL-33 and its translocation to cytoplasm, where it mediates T helper 2 cells (Th2)-type immune responses through autocrine or paracrine engagement of membrane-bound ST2 receptors on adjacent cells (Fig. 5D) [22]. Initially, Astrocyte-secreted supernatant showed increased levels of IL-33 following MPP^+^ stimulation, as detected by ELISA (Fig.5E). Additionally, nuclear and cytoplasmic proteins of astrocytes were separated, and the efficiency of separation was confirmed (Fig. S8J). Increased protein levels of IL-33 in the cytoplasm were found, while the levels of IL-33 in the nucleus were reduced (Fig. 5F, G). These findings suggest that nuclear IL-33 is depleted and actively secreted into the cytoplasm, potentially promoting extracellular inflammatory regulation after MPP^+^ stimulation.

### ***Il33*** Overexpression Promotes A2 Polarization of Astrocytes Induced by MPP^+^

**Fig. 6:**
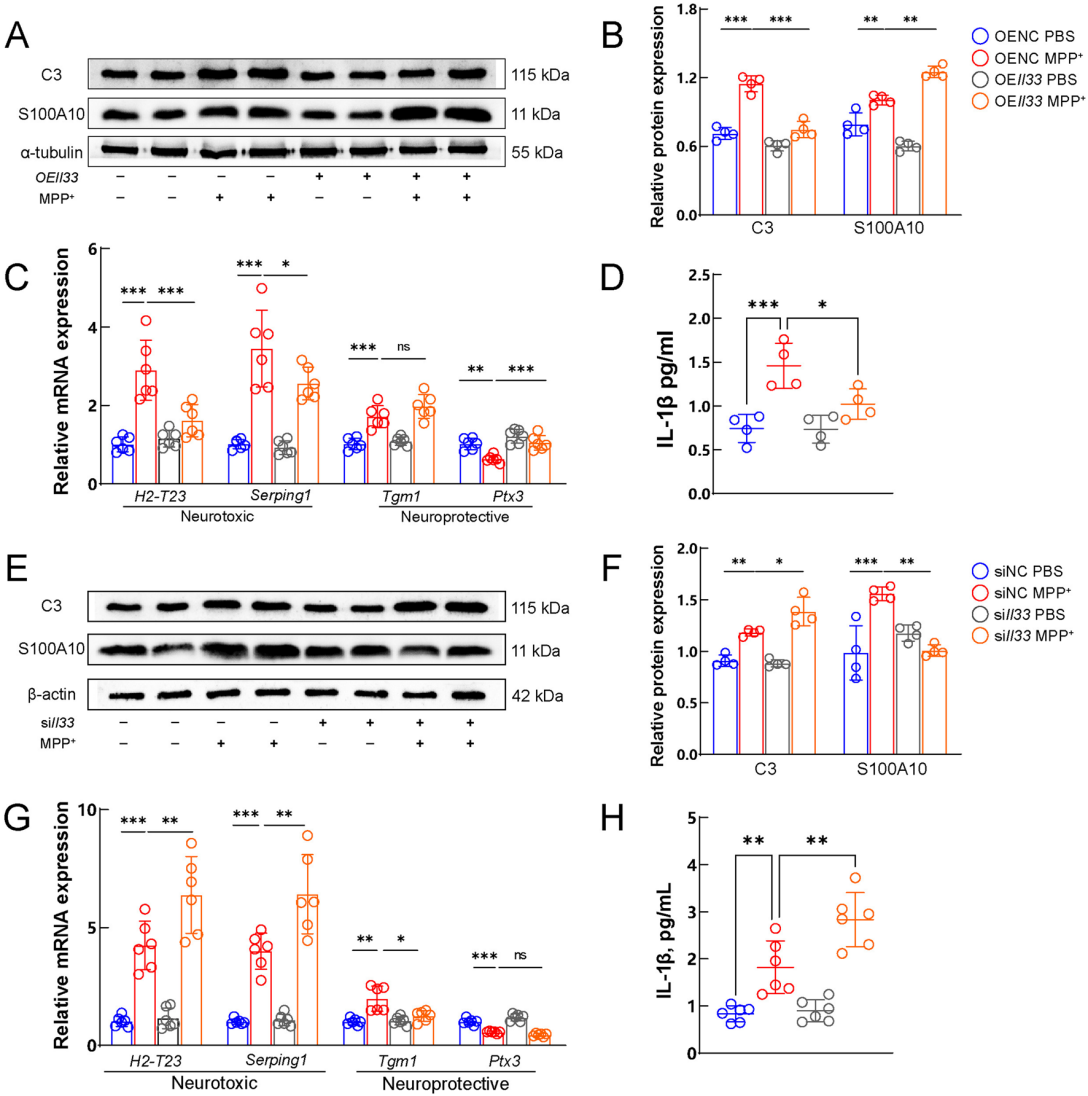
Interference with *Il33* expression changes the phenotypic transformation of astrocytes. **A, B** Protein expression of C3 and S100A10 in astrocytes assessed by WB (**A**) and quantitative analyses (**B**) among groups, *n =* 4 per group; **C** qPCR analyses of mRNA expression of *H2T23*, *Serping1*, *Tgm1* and *Ptx3*, *n =* 4 per group; **D** Quantification of IL-1β levels in astrocyte-secreted supernatant detected by ELISA, *n =* 4 per group; **E, F** Protein expression of C3 and S100A10 in astrocytes assessed by WB (**E**) and quantitative analyses (**F**) among groups, *n =* 4 per group; **G** qPCR analyses of mRNA expression of *H2T23*, *Serping1*, *Tgm1* and *Ptx3*, *n =* 4 per group; **H** Quantification of IL-1β levels in astrocyte-secreted supernatant detected by ELISA, *n =* 4 per group; Significances were assessed using two-way ANOVA. *P < 0.05, **P < 0.01, ***P < 0.001, ****P < 0.0001.

An *Il33*-overexpressing (OE*Il33*) *in vitro* model was established (Fig. S9A), and it was confirmed successfully (Fig. S9B–F). Meanwhile, the secreted levels of IL-33 were elevated after MPP^+^-stimulated (Fig. S9G), indicating that overexpressing IL-33 not only increased IL-33 expression but also enhanced its cytoplasmic secretion. Compared to the control group, OE*Il33* astrocytes exhibited lower levels of C3 and increased levels of S100A10 after MPP^+^ stimulation (Fig. 6A, B). qPCR analysis further showed that neurotoxic markers *H2-T23* and *Serping1* were significantly downregulated, while A2 markers *Tgm1* and *Ptx3* were upregulated (Fig. 6C). These results suggest that *Il33* overexpression promotes astrocyte transition to a neuroprotective phenotype. Pro-inflammatory markers such as *Tnf, Il6*, and *Ccl2* were raised in MPP^+^-stimulated OE*Il33* astrocytes (Fig. S9H), while anti-inflammatory markers, including *Bdnf*, *Tgfb1*, and *Vegf,* were declined (Fig. S9I). Moreover, IL-1β secretion from OE*Il33* astrocytes was significantly lower (Fig. 6D).

### ***Il33*** Knockdown Aggregates A1 Polarization of Astrocytes Induced by MPP^+^

Next, we employed *Il33* siRNA to knock down the expression of IL-33. Successful establishment of the *Il33*-knockdown *in vitro* model was verified (Fig. S9J–N). Besides, IL-33 secretion was significantly reduced in si*Il3*3 astrocytes (Fig. S9O). The levels of C3 protein were elevated in MPP^+^-stimulated si*Il33* astrocytes compared to non-knockdown groups, while the S100A10 levels were decreased (Fig. 6E, F). qPCR results showed significant upregulation of *H2-T23* and *Serping1*, and downregulation of *Tgm1* and *Ptx3* (Fig. 6G). Additionally, *Tnf*, *Il6*, and *Ccl2* were elevated in MPP^+^ si*Il33* astrocytes (Fig. S9P), while *Bdnf*, *Tgfb1*, and *Vegf* were decreased (Fig. S9Q). IL-1β secretion in si*Il33* astrocytes after MPP^+^ stimulation was significantly increased compared to non-knockdown groups (Fig. 6H), further confirming that *Il33* knockdown exacerbates inflammatory responses.

### RNA-seq Screening for Inflammation-related Hub Genes

**Fig. 7:**
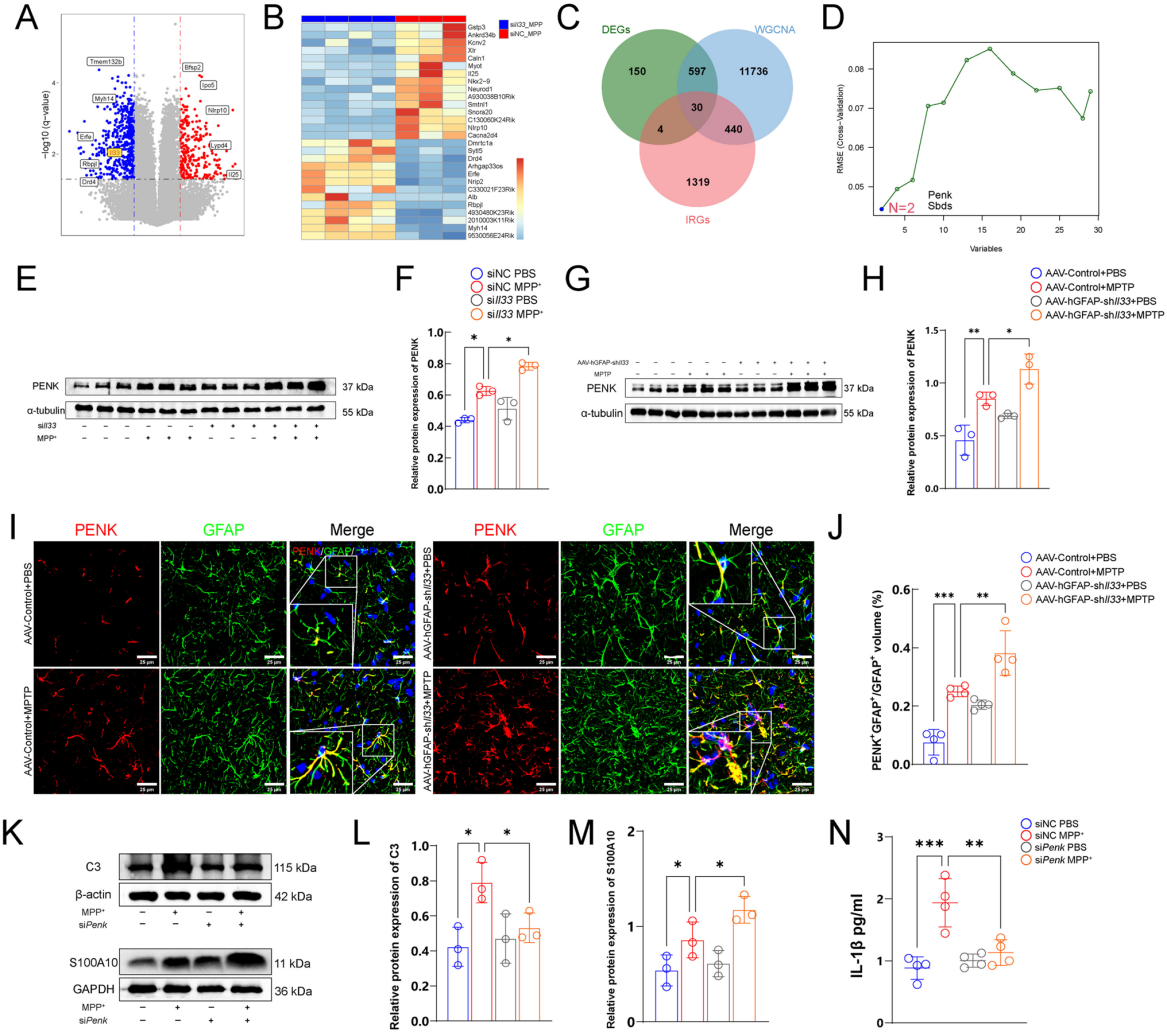
Hub gene identified from RNA-seq and validated *in vivo* and in *vitro*. **A** Volcano plot illustrating the distribution of upregulated and downregulated DEGs; **B** Heatmap depicting the top 15 most significantly upregulated and downregulated DEGs; **C** Venn plot displaying the overlaps among DEGs, IRGs, and molecule genes identified by WGCNA; **D** SVM-RFE model of the merged genes; **E, F** Protein expression of PENK in astrocytes assessed by WB (**E**) and quantitative analyses (**F**) among groups, *n =* 3 per group; **G, H** Protein expression of PENK in the SN assessed by WB (**G**) and quantitative analyses (**H**) in the SN among groups, *n =* 3 per group; **I** Representative confocal images of PENK (red) and GFAP (green) in the SN, scale bar = 25 μm; **J** Quantitative analysis of the ratio of PENK^+^GFAP^+^ to GFAP^+^ area in the SN, *n =* 4 per group; **K-M** Protein expression of C3 and S100A10 in astrocytes assessed by WB (**K**) and quantitative analyses (**L**, **M**) among groups, *n =* 3 per group; **N** Quantification of IL-1β levels in astrocyte-secreted supernatant detected by ELISA, *n =* 4 per group; Significances were assessed using two-way ANOVA. *P < 0.05, **P < 0.01, ***P < 0.001, ****P < 0.0001.

RNA-seq was performed on 4 siNC-MPP^+^ and 3 si*Il33*-MPP^+^ samples (Table S8). Quality control confirmed that all samples met the requirements for subsequent analyses (Fig. S10A–C). A total of 781 DEGs were identified, with 276 upregulated and 505 downregulated DEGs. Data visualization, including volcano plots (Fig. 7A) and cluster heatmaps of the top 15 DEGs (Fig. 7B), was performed to summarize the findings. GO and KEGG analyses were performed, with pathways visualized by sorting enrichment factors from high to low (Fig. S10D, E). GSEA revealed significant enrichment in pathways related to immune regulation, energy metabolism, and neurodegeneration (Fig. S10F). The PPI network consisted of 551 nodes and 138 edges (Fig. S10G). WGCNA identified 9 modules after dynamic tree cut merging, with the turquoise module showing significant inter-group divergence (Fig. S10H). Then, we intersected DEGs, WGCNA turquoise module genes, and IRGs retrieved from GeneCards. Venn diagram revealed 29 overlapping genes, including 13 upregulated and 16 downregulated (Fig. 7C). GO, KEGG, and PPI were performed based on the hub genes (Fig. S10I–K). To validate the robustness of the hub gene, SVM-RFE was applied to the 29 candidates. Machine learning identified two signature genes: SBDS ribosome maturation factor (SBDS) and preproenkephalin (PENK) (Fig. 7D). Specifically, *Penk* was significantly upregulated in *Il33*-knockdown astrocytes (Fig. S11A), while *Sbds* was downregulated (Fig. S11B).

### Expression of PENK in *In vivo* and *In vitro* Models

Following the identified hub genes, literature reviews highlighted a potential role for PENK in PD pathogenesis. PENK encodes enkephalin precursors, which are proteolytically processed into bioactive peptides that mediate neuro-glial interactions and neuroimmune crosstalk [23, 24]. The expression level of PENK was verified both *in vivo* and *in vitro*. After MPP^+^ stimulation, *Penk* and its receptor, *Opioid growth factor receptor* (*Ogfr*), were significantly upregulated, while Apelin (*Apln*) was downregulated (Fig. S11C). Elevated PENK in *si*Il33 astrocytes after MPP^+^ stimulation was detected (Fig. 7E, F), suggesting that IL-33 may influence astrocyte function by regulating PENK and its receptor expression. The localization and expression level of PENK were further detected in MPTP models with astrocyte-specific *Il33* knockdown. WB results confirmed that PENK protein levels were significantly increased after *Il33* knockdown (Fig. 7G, H). PENK was predominantly co-localized with GFAP (Fig. 7I), while not with IBA1 (Fig. S11D). Moreover, PENK in astrocytes of MPTP mice was significantly higher than in the Controls. The ratio of PENK^+^GFAP^+^ area to GFAP^+^ in *Il33*-knockdown MPTP mice was further increased (Fig. 7I, J). These results indicate that the IL-33 deficiency of astrocytes exerts a negative regulatory effect on PENK expression.

### *Penk* Knockdown Ameliorates Neurotoxic and ERK/MAPK Signaling Activation

siRNA-mediated knockdown of *Penk* of astrocytes was performed, and successful establishment of the model was confirmed (Fig. S11E–I). MPP^+^-stimulated si*Penk* astrocytes had significantly reduced C3 and increased S100A10 levels compared to non-knockdown groups (Fig. 7K-M). Furthermore, downregulation of *H2-T23* and *Serping1* (Fig. S11J), and upregulation of *Tgm1* and *Ptx3* (Fig. S11K) were observed, confirming that *Penk* knockdown promotes the transition of astrocytes to a neuroprotective phenotype. Significant decrease of *Tnf*, *Il6*, and *Ccl2* (Fig. S11L), as well as an increase of *Bdnf*, *Tgfb1*, and *Vegf* (Fig. S11M) were detected in MPP^+^-stimulated si*Penk* astrocytes. IL-1β secretion in si*Penk* astrocytes was also lower than in normal expression groups (Fig. 7N).

**Fig. 8:**
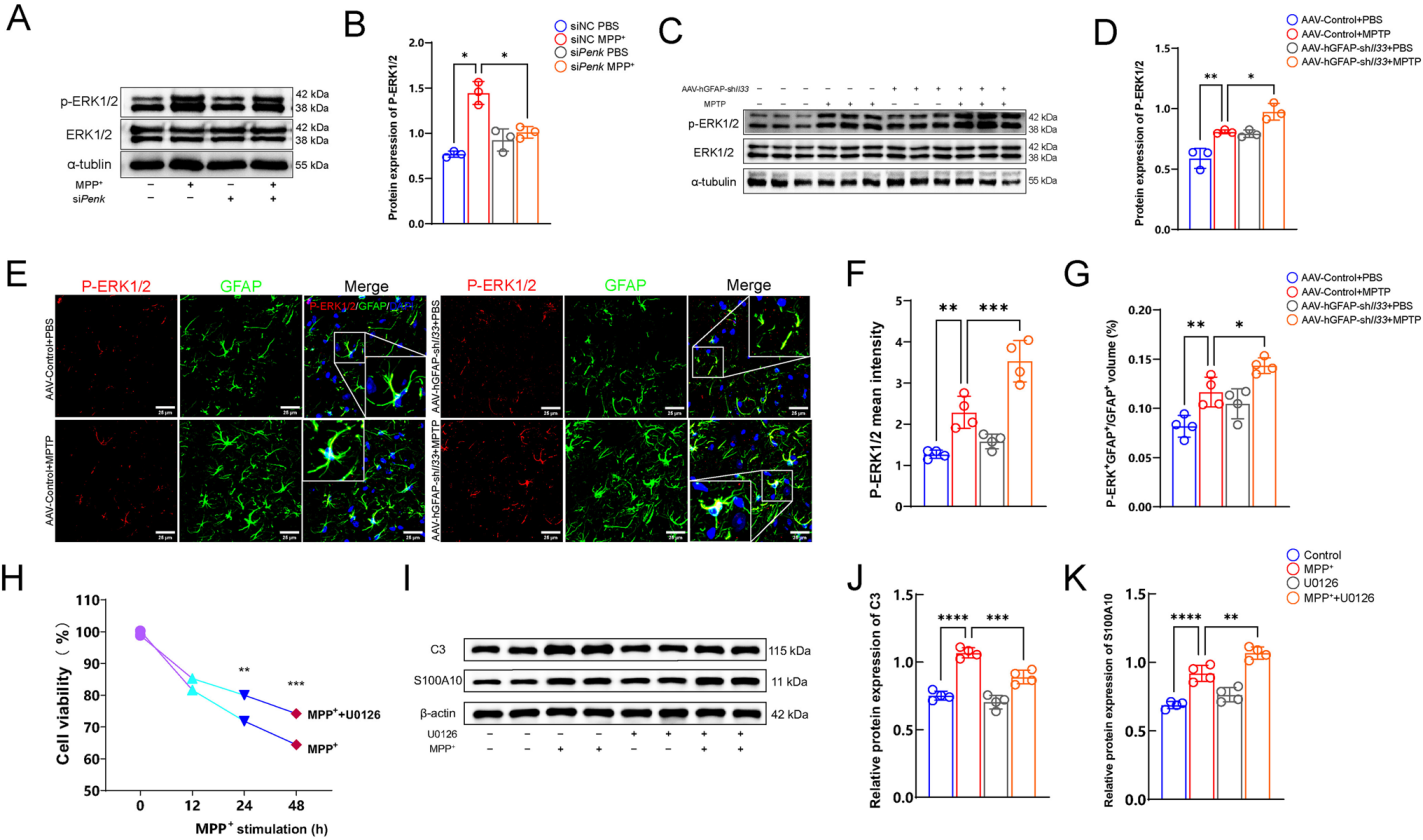
The role of PENK-ERK/MAPK pathway validated *in vivo* and in *vitro*. **A, B** Protein expression of p-ERK1/2 in astrocytes assessed by WB (**A**) and quantitative analyses (**B**) among groups, *n =* 3 per group; **C, D** Protein expression of p-ERK1/2 in the SN assessed by WB (**C**) and quantitative analyses (D) among groups, *n =* 3 per group; **E** Representative confocal images of p-ERK1/2 (red) and GFAP (green) in the SN, scale bar = 25 μm;** F** Quantification of p-ERK1/2 average intensity in groups, *n =* 4 per group; **G** Quantitative analysis of the ratio of p-ERK1/2^+^GFAP^+^ to GFAP^+^ area in the SN, *n =* 4 per group; **H** Cell viability of MPP^+^- and U0126-treated astrocytes assessed by CCK-8; **I–K** Protein expression of C3 and S100A10 in astrocytes assessed by WB (**I**) and quantitative analyses (**J**, **K**) among groups, *n =* 3 per group; Significances were assessed using two-way ANOVA. *P < 0.05, **P < 0.01, ***P < 0.001, ****P < 0.0001.

Emerging evidence implicates PENK in the activation of the ERK/MAPK and P38/MAPK signaling axes [23, 25]. Next, we examined the activation of key initiating genes in these pathways. Significant upregulation of *Erk1*, *Erk2*, and *P38* mRNA levels was detected in MPP^+^-stimulated astrocytes. After the *Penk* knockdown, *Erk1* and *Erk2* expressions were significantly downregulated (Fig. S12A), suggesting that PENK may regulate astrocyte function by activating the ERK/MAPK signaling. The ratio of phosphorylated-ERK1/2 (p-ERK1/2) to ERK1/2 in si*Penk* astrocytes was significantly lower than in non-knockdown groups (Fig. 8A, B). Further validation was conducted in the MPTP model with astrocyte-specific *Il33* knockdown. Protein levels of p-ERK1/2 in the SN of MPTP mice were higher than in the Controls, and p-ERK1/2 levels were further increased in MPTP mice with specific *Il33* knockdown (Fig. 8C, D). The average fluorescence intensity of p-ERK1/2 and the ratio of p-ERK1/2^+^GFAP^+^ to GFAP^+^ area were abnormally increased in *Il33* knockdown MPTP mice (Fig. 8E–G). These findings confirm that IL-33 deficiency of astrocytes is closely associated with the activation of the ERK/MAPK pathway.

### ERK/MAPK Inhibitor Ameliorates Neurotoxic and Inflammatory Response

Astrocytes were pretreated with 10 μmol/L U0126 prior to MPP^+^ stimulation, and inhibition efficiency was verified by WB (Fig. S12B, C). CCK-8 assays demonstrated that inhibition of the ERK/MAPK pathway reversed astrocyte viability relative to the MPP^+^-only group (Fig. 8H). Compared to the untreated group, U0126-treated astrocytes exhibited significantly reduced C3 levels and elevated S100A10 levels (Fig. 8I-K). Furthermore, downregulation of *H2-T23* and *Serping1* (Fig. S12D), and upregulation of *Tgm1* (Fig. S12E) were detected, confirming that ERK inhibition promotes astrocytes transition to a neuroprotective phenotype. Additionally, U0126-treated astrocytes showed significant decreases in *Tnf* and *Il1β* levels, as well as an increase in *Tgfb1* levels (Fig. S12F).

## Discussion

The present study provides compelling evidence that IL-33 plays a pivotal role in modulating astrocyte activation and neuroinflammation in PD. By integrating clinical observations with *in vivo* and *in vitro* experiments, we demonstrated that IL-33 regulates the phenotypic transformation of reactive astrocytes, influences disease progression, and holds therapeutic potential. Additionally, plasma IL-33 and sST2 may serve as valuable biomarkers for the diagnosis and monitoring of PD.

IL-33 is recognized as a dual-function cytokine that exerts either pro- or anti-inflammatory effects and can act as a regular cytokine or nuclear transcription factor. Its role is highly context-dependent, varying with the experimental model, disease type, and target cell type [26]. Accumulating evidence indicates that IL-33 primarily plays a neuroprotective role in diverse neurological diseases, and the IL-33/ST2 pathway shows promise for novel biomarker discovery and as a therapeutic target in these conditions [27]. In AD, *Il33* knockout exacerbates Tau hyperphosphorylation and cognitive impairment [28], while exogenous IL-33 supplementation reduces amyloid-beta deposition by enhancing microglial phagocytosis [29]. In multiple sclerosis, IL-33 ameliorates experimental autoimmune encephalomyelitis via regulatory T cells but may also enhance chronic neuroinflammation under certain conditions [30].

The neuroprotective role of IL-33 in PD aligns with its functions in other neurodegenerative diseases, but it also exhibits PD-specific characteristics. A prior work and our experiment identify that IL-33 deficiency exacerbates DA neuron loss and neuroinflammation activation, and rIL-33 alleviates PD pathology, confirming the neuroprotective role of IL-33 in PD [31]. Recent work has focused on IL-33’s regulation of microglial activation via the NF-κB pathway in PD, but the cell-type-specific function of IL-33 remained unaddressed. Our experiments have discovered that the PD-specific function of IL-33 is distinguished by its regulation of astrocyte polarization. Notably, IL-33 exerts dual roles in PD progression. In the acute phase of MPTP exposure, transient IL-33 upregulation acts as a compensatory mechanism to initiate protective astrocyte polarization and Th2 responses [22]. However, long-term neurotoxic stress depletes IL-33, reducing the reserve of neuroprotective A2-type astrocytes and exacerbating PD progression [32]. This dual role aligns with IL-33’s role as an “alarmin”: protective in acute injury but critical for maintaining tissue homeostasis in chronic degenerative processes [33]. Cellular localization further underlies IL-33’s functional duality in PD. As a classical nuclear chromatin-binding protein, IL-33 is primarily localized in astrocyte nuclei under physiological conditions, where it maintains transcriptional homeostasis [34]. Upon cellular injury, IL-33 is released extracellularly to act as an alarmin, activating ST2-mediated signaling in adjacent astrocytes and triggering protective cascades [22]. Our data show reduced nIL-33 levels in MPTP-induced PD mice, suggesting neurotoxins disrupt both IL-33’s nuclear homeostatic function and its extracellular protective effects. This observation highlights the need for extracellular IL-33 supplementation in late-stage PD.

Astrocyte-mediated inflammation is recognized key contributor to PD pathogenesis, serving as a major amplifier of neuroinflammation [35]. Activated microglia release pro-inflammatory mediators that induce astrocytes to transition into the pro-inflammatory A1 phenotype, further exacerbating the release of inflammatory cytokines [36, 37]. While some studies suggest astrocytes exert neuroprotective effects in the early stage of injury, DA neuronal damage disrupted the injury-repair balance, shifting astrocytes toward a neurotoxic phenotype [38]. Our data support that injection of rIL-33 into the SN reduced A1 astrocyte markers and increased A2 markers, indicating IL-33 promotes the transition of astrocytes from neurotoxic to a neuroprotective state. We further identified astrocytes as the primary source of IL-33. Specific knockdown of astrocyte-derived IL-33 blocked its secretion, exacerbating DA neuron loss, motor symptoms, and neurotoxic astrocyte polarization. This underscores the critical role of IL-33 in maintaining astrocyte-mediated neuroprotection, which is consistent with prior findings [10, 39]. *In vitro*, *Il33* overexpression boosts astrocyte IL-33 secretion, promoting the formation of A2 astrocytes and reducing inflammation. Conversely, *Il33* knockdown increased A1 astrocytes and worsened inflammation. It is important to note that while the A1/A2 dichotomy provides a foundational framework for understanding astrocyte heterogeneity, it has been criticized for oversimplifying the complexity of reactive astrocyte [36]. Recent studies have highlighted that astrocytes exhibit transcriptional and functional diversity beyond the A1/A2 paradigm, with intermediate phenotypes observed in neurodegenerative models [40]. Single markers are insufficient to fully capture the transcriptional complexity of astrocytes; future single-cell RNA sequencing studies will likely unravel more nuanced IL-33-regulated astrocyte subpopulations. Former studies have also shown that astrocytes in the CNS regulate microglial function by releasing IL-33, which plays a key role in brain maturation [11, 12]. Additionally, astrocyte-released IL-33 amplifies microglial function of neural synapses and remodels neural circuits during CNS development [11]. Collectively, these findings support IL-33 as a potential PD therapeutic target for PD, as it exerts neuromodulatory effects by inhibiting neuroinflammation and preventing astrocyte transformation toward neurotoxic phenotypes.

Mechanistically, RNA-seq analysis of *Il33*-knockdown astrocytes highlighted the critical role of PENK in IL-33-regulated astrocyte function. PENK is an endogenous opioid peptide (enkephalins) and is involved in pain regulation and neuroprotection [41]. It is highly expressed in astrocytes and lymphocytes [41, 42]. Previous studies have shown that the loss of nigrostriatal DA neurons reduces dendritic spines on striatal medium spiny neurons, and PENK is primarily produced by these neurons under DA signaling [23]. In PD models, striatal PENK is upregulated and linked to involuntary movements, and its levels remain elevated after levodopa treatment, positioning PENK as a potential target related to PD motor symptoms [24]. Relevant to astrocyte biology, prior work has demonstrated that arachidonic acid promotes PENK expression by activating the p38 and ERK pathways, leading to the phosphorylation of CREB [43]. Another study reported that IL-1β and TNF-α enhance PENK expression [44]. Our *in vivo* and *in vitro* experiments extend these findings that *Il33* knockdown increased astrocyte PENK levels, while si*Penk* inhibited MPP⁺-induced ERK1/2 phosphorylation. It suggests PENK drives astrocyte neurotoxicity via ERK/MAPK pathway activation. Furthermore, *Il33* knockdown elevated p-ERK1/2 levels, and this effect was reversed by si*Penk*, confirming that IL-33 regulates the ERK/MAPK pathway in a PENK-dependent manner.

Plasma IL-33 and sST2 demonstrate clear utility for improving PD diagnosis, monitoring disease progression, and guiding treatment strategies, aligned with our findings and recent evidence. Notably, plasma sST2 is abnormally elevated in both AD and PD patients, correlating with deficits in working memory, executive function, and visuospatial ability [15, 29, 45]. In contrast, plasma IL-33 does not decrease significantly in early-stage PD, likely reflecting a compensatory response to neuroinflammation. It only declines as the disease progresses, and this compensatory balance is disrupted. The role of IL-33 in early PD and its depletion in late stages may inform stage-specific therapeutic strategies, such as targeted IL-33 supplementation in advanced PD to restore neuroprotective effects.

For motor symptoms, reduced plasma IL-33 and elevated sST2 reflect exacerbated immune dysregulation, a process that impairs DA neurons' survival, and thereby worsens motor symptoms. Specifically, lower IL-33 levels may diminish neuronal protection, contributing to progressive DA neuron death [22]. Meanwhile, increased sST2 leads to competitive binding with IL-33 receptors, further exacerbating neuroinflammation. For non-motor symptoms, IL-33 regulates neuronal proliferation, differentiation, and synaptic plasticity, which are processes critical for cognitive function [11]. Increased sST2 levels are associated with neuroinflammation in the cerebral cortex and hippocampus, which disrupts cognition [22]. Reduced IL-33 also reflects underlying neuroinflammation and neurotransmitter imbalance, potentially contributing to sleep disturbances [46]. Additionally, we observed significant correlations between sST2 levels and blood biochemical indices, iron metabolism, and the midbrain area. These findings suggest that sST2 could serve as an auxiliary diagnostic marker for PD, potentially improving diagnostic accuracy. For diagnosis, dynamic changes of IL-33 and sST2 support these as accessible, non-invasive biomarkers for PD diagnosis and progression monitoring. Therapeutically, preclinical data highlight IL-33’s neuroprotective role in PD. It suggests that targeted IL-33 supplementation, especially in late-stage PD where endogenous IL-33 is depleted, could be a viable therapeutic strategy.

This study has several advantages that should be addressed. Our study prioritizes astrocyte phenotypic transformation, employing astrocyte-specific *Il33* knockdown to demonstrate that astrocyte-derived IL-33 restricts neurotoxic astrocytes and promotes neuroprotective astrocytes. Mechanistically, we identified the novel PENK-ERK/MAPK pathway as a mediator of IL-33’s effects on astrocyte polarization. Clinically, current research further validates plasma IL-33 and sST2 as PD biomarkers. However, this study has limitations. First, the clinical data are cross-sectional, which limits the ability to capture the dynamic changes in plasma IL-33 and sST2 levels during PD progression. Long-term follow-up of subjects is needed to provide stronger evidence for early diagnosis and monitoring. Additionally, using *Il33* or *Il1rl1* knockout mice would allow for a more detailed exploration of the mechanism underlying the IL-33/ST2 signaling pathway in PD. Finally, while this study found that IL-33 regulates astrocytes via the PENK-ERK/MAPK pathway, the specific molecular interactions and roles of key molecules in this pathway remain unclear. Further studies are required to fully elucidate the role of IL-33 in PD pathogenesis.

In summary, IL-33 exerts a neuroprotective role in PD by regulating the phenotypic transformation of astrocytes. Mechanistic exploration has revealed that IL-33 regulates astrocyte function and inflammatory responses through the PENK-ERK/MAPK pathway. Additionally, plasma IL-33 and sST2 can serve as potential biomarkers and indicators for monitoring disease progression in PD.

## Supplementary Information

Below is the link to the electronic supplementary material.

Supplementary file1 (PDF 1617 KB)

## Data Availability

The datasets generated during and/or analyzed during the current study are available from the corresponding author on reasonable request.
