# Supplementary material for: IL-33 Regulates the Phenotypic Transformation of Reactive Astrocytes via PENK-ERK/MAPK Pathway in Parkinson’s Disease
Source: Neurosci Bull. 2026 Jan 3;42(7):1551–68. doi: 10.1007/s12264-025-01566-2 (PMC13388577; doi:10.1007/s12264-025-01566-2)
Supplement: Supplementary file 1 — Supplementary file1 (PDF 1617 KB) [file 12264_2025_1566_MOESM1_ESM.pdf]

Supplementary Materials

CONTENT

TITLE	PAGE
Supplementary Materials and Methods	1-3
Table S1. The primary antibodies used for immunofluorescence	4
Table S2. The primer sequences for quantitative real-time PCR	5
Table S3. The primary antibodies used for the Western blot	6
Table S4. The characteristics of enrolled participants	7
Table S5. The accuracy of plasma sST2 in the diagnosis of PD in the ROC curve	8
Table S6. The characteristics of PD patients stratified by H&Y stages	9
Table S7. The association of plasma IL-33 or sST2 levels with PD features	10
Table S8. The sample characteristics of astrocytes in the RNA seq.	11
Fig. S1. Flowchart of included participants.	12
Fig. S2. Associations of plasma IL-33 and sST2 levels with PD symptoms	13
Fig. S3. The expression of IL-33 and ST2 in the substantia nigra.	14
Fig. S4. rIL-33 supplementation ameliorates DA neuron loss and neuroinflammation in the MPTP mouse model.	15
Fig. S5. rIL-33 supplementation improves microglial neuroinflammation in the MPTP mouse model.	16
Fig. S6. Astrocyte-specific *Il33* knockdown aggravates PD-like symptoms and neuroinflammation in the MPTP mouse model.	17
Fig. S7. Astrocyte-specific Il33 knockdown aggravates microglial neuroinflammation in the MPTP mouse model.	18
Fig. S8. Dynamic expression of A1/A2 markers and IL-33 in astrocytes.	19
Fig. S9. The validation of the astrocytes model with Il33 expression and the phenotypic transformation of astrocytes.	20-21
Fig. S10. Identification of hub gene in si*Il33* astrocytes	22
Fig. S11. The expression and role of PENK in astrocytes	23-24
Fig. S12. The role of PENK-ERK/MAPK pathway in astrocytes.	25

Supplementary Materials and Methods

Clinical assessments

The Movement Disorder Society Unified Parkinson's Disease Rating Scale (MDS-UPDRS) Parts I, II, and III, and the modified Hoehn-Yahr (H&Y) assessed the motor and non-motor symptoms. Cognitive function was evaluated using the Mini-Mental State Examination (MMSE) and Montreal Cognitive Assessment (MoCA), emotional states by the Hamilton Depression Rating Scale (HAMD) and Hamilton Anxiety Rating Scale (HAMA), and sleep disturbances using the Pittsburgh Sleep Quality Index (PSQI).

Behavioral test

Motor function in mice was evaluated using a rotarod treadmill (Sansbio, China). Prior to the formal test, all mice underwent training on the rotarod at a constant speed of 10 rpm for 10 min over 3 days. Six days post-MPTP treatment, the formal rotarod test was conducted with a speed increasing uniformly from 5 to 40 rpm over 300 s, during which the latency to fall was recorded. Mice that remained on the rod for the full 5 min were recorded as having a latency of 300 s.

The pole test was also adopted to evaluate the motor function of mice. The apparatus consisted of a 55 cm high, 1 cm diameter wooden pole, wrapped with gauze to prevent slipping, with a wooden ball at the top. After acclimatization, the mice underwent three pre-training sessions with the pole which all animals would turn head-down upon being placed on the ball. During the pole test, the total time taken for a mouse to descend from the top to the bottom was measured. These tests were repeated three times for each mouse, and the average time was recorded.

The open field test (OFT) was employed to assess the behavioral and emotional alterations of mice in a new environment, including spontaneous motor activity and anxiety-related behavior. The environment for the OFT was a 30 × 30 × 35 box, and its bottom was divided into 9 squares of the same size. No pre-training or adaptation was necessary for the animals. The mice were placed at the edge of the box, and their movement trajectories were recorded for 5 minutes. ANY-MAZE (Version 7.16, Stoelting, Wood Dale, IL) was used to analyze behavioral indicators such as total distance, immobility time, and the time spent in the central area.

Brain tissue preparation

All mice were sacrificed one day after the behavioral tests, and their brain tissues were processed for subsequent experiments and analyses. For frozen sections, mice were anesthetized

with isoflurane and perfused transcardially with 30 mL of precooled PBS, followed by 20 mL of 4% paraformaldehyde (PFA). Subsequently, the brains were harvested and post-fixed in 4% PFA for 24 hours. After fixation, the brains were dehydrated in a PBS solution containing 30% sucrose until the samples sank to the bottom. Coronal sections (12 μm thick) of SN pars compacta (SNpc) were then cut on a freezing slicer (Thermo Fisher Scientific, USA) and mounted on coated slides for analysis. For SN tissues, anesthetized mice were perfused with 40 mL of precooled PBS before the brains were quickly sectioned at 1 mm thickness in the coronal plane using a precooled mouse brain matrix (RWD Life Science, China). The SN tissue was segmented according to 'The Mouse Brain in Stereotaxic Coordinates, third edition', snap-frozen in liquid nitrogen-cooled isopentane, and stored at −80°C.

Cell viability assay

Cell viability was assessed using the CCK-8 kit (Yeasen Biotechnology). Briefly, 100 μL of medium containing 10 μL of CCK-8 reagent was added to each well and incubated at 37 °C for 4 h. Absorbance was measured at 450 nm using a microplate reader (Thermo Fisher Scientific, Waltham, MA).

Enrichment analyses

Gene Ontology (GO) includes three categories: molecular function (MF), cellular component (CC), and biological process (BP), which comprehensively describe gene functions. Combined with Kyoto Encyclopedia of Genes and Genomes (KEGG) analysis, the enrichment of DEGs in signaling pathways was evaluated. *P*-values of GO functional annotations and KEGG enrichment pathways were ranked, and the top 10 GO terms and KEGG pathways were selected. Gene Set Enrichment Analysis (GSEA) is an advanced functional method that uses pre-defined gene sets to evaluate differential expression between groups and test for enrichment at the top/bottom of ranked lists. Compared to traditional KEGG or GO analysis, GSEA more accurately compares gene up-/down-regulation differences in the same pathway between groups, providing an effective tool for dealing with complex biological phenomena.

PPI, WGCNA, and machine learning

Protein-protein interaction (PPI) analysis of DEGs was performed using the DAVID database (http://david.abcc.ncifcrf.gov/), constructing interaction networks of DEG-encoded proteins. Nodes represented proteins, and edges represented interactions. Weighted gene co-expression network

analysis (WGCNA) constructed weighted networks based on correlation weights in gene co-expression matrices for multi-gene enrichment analysis. Venn diagrams identified common genes among DEGs, WGCNA, and inflammatory-related genes (IRGs) to determine key genes. A support vector machine-recursive feature elimination (SVM-RFE) model was constructed to identify disease-related key genes through machine learning.

Analysis tools

Data processing was carried out using the Bioconductor platform (version 3.6) of R software. The screening of DEGs was based on the limma package. The volcano plot was drawn using the ggplot2 package, and the clustering heatmap was created using the pheatmap package. The clusterprofiler package was used for GO, KEGG, and GSEA analyses. The WGCNA package was used to construct the co-expression network of DEGs. The e1071, kernlab, and caret packages were used for SVM-RFE analysis.

Table S1. The primary antibodies used for immunofluorescence.

Antibodies	Source	Cat. No.	Host	Dilution
C3	Abcam	ab11862	Rat	1:100
C3d	R&D	AF2655	Goat	1:200
ERK1/2	Proteintech	11257-1-AP	Rabbit	1:400
GFAP	CST	GA5	Mouse	1:750
GFAP	Proteintech	16825-1-AP	Rabbit	1:750
GFAP	Abcam	ab302644	Goat	1:750
IL-33	Abcam	ab187060	Rabbit	1:200
IL-33	R&D	AF3626	Goat	1:200
IBA1	Wako	019-19741	Rabbit	1:500
IBA1	Abcam	ab289874	Goat	1:500
NeuN	CST	D4G4O	Rabbit	1:200
OLIG2	R&D	AF2418	Goat	1:200
PENK	Abclonal	A6302	Rabbit	1:200
P-ERK1/2	Proteintech	80031-1-RR	Rabbit	1:200
S100A10	Proteintech	15146-1-AP	Rabbit	1:200
ST2	Abcam	ab25877	Rabbit	1:200
TH	Novus Biologicals	NB300-110	Sheep	1:500
TH	Santa	sc-25269	Mouse	1:100
TH	Gentex	GTX113016	Rabbit	1:500

Table S2. The primer sequences for quantitative real-time PCR.

Gene	Direction	Primer 5′-3′
Actb	F	TCCGGCACTACCGAGTTATC
	R	GATCCGGTGTAGCAGATCGC
Apln	F	TGAATCTGAGGCTCTGCGTG
	R	ACATCAGTGGCACTCCACAA
Bdnf	F	TCATACTTCGGTTGCATGAAGG
	R	AGACCTCTCGAACCTGCCC
Ccl2	F	TTAAAAACCTGGATCGGAACCAA
	R	GCATTAGCTTCAGATTTACGGGT
C3	F	CCAGCTCCCCATTAGCTCTG
	R	GCACTTGCCTCTTTAGGAAGTC
H2-T23	F	ACAGTCCCGACCCAGAGTAG
	R	CCACGTAGCCGACAATGATGA
Il1rl1	F	TGACACCTTACAAAACCCGGA
	R	AGGTCTCTCCCATAAATGCACA
Il33	F	TCCAACTCCAAGATTTCCCCG
	R	CATGCAGTAGACATGGCAGAA
Erk1	F	TCCGCCATGAGAATGTTATAGGC
	R	GGTGGTGTTGATAAGCAGATTGG
Erk2	F	CAGGTGTTCGACGTAGGGC
	R	TCTGGTGCTCAAAAGGACTGA
Il6	F	CCTACCCCAATTTCCAATGCT
	R	TATTTTCTGACCACAGTGAGGAAT
Ogfr	F	CTGACTCTGACACTACAGGAACC
	R	GCCTCAATCCTTGCCAACTC
Penk	F	GAGAGCACCAACAATGACGAA
	R	TCTTCTGGTAGTCCATCCACC
P38	F	GGCTCGGCACACTGATGAT
	R	TGGGGTTCCAACGAGTCTTAAA
Ptx3	F	CCTGCGATCCTGCTTTGTG
	R	GGTGGGATGAAGTCCATTGTC
Serping1	F	TAGAGCCTTCTCAGATCCCGA
	R	ACTCGTTGGCTACTTTACCCA
S100a10	F	TGGAAACCATGATGCTTACGTT
	R	GAAGCCCACTTTGCCATCTC
Tgfb1	F	CTCCCGTGGCTTCTAGTGC
	R	GCCTTAGTTTGGACAGGATCTG
Tgm1	F	TCTGGGCTCGTTGTTGTGG
	R	AACCAGCATTCCCTCTCGGA
Tnf	F	CCCTCACACTCAGATCATCTTCT
	R	GCTACGACGTGGGCTACAG
Vegf	F	GCGACCCACACGTCAAACTA
	R	TCCCTTGATAGACACAACTCCTC

Table S3. The primary antibodies used for the Western blot.

Antibodies	Source	Cat. No.	Host	Dilution
α-tubulin	Proteintech	11224-1-AP	Rabbit	1:5000
β-actin	Abclonal	AC004	Mouse	1:5000
C3d	R&D	AF2655	Goat	1:2000
ERK1/2	Proteintech	11257-1-AP	Rabbit	1:5000
GAPDH	HUABIO	ET1601-4	Mouse	1:5000
GFAP	Proteintech	16825-1-AP	Rabbit	1:5000
Histone H3	Abclonal	A17562	Rabbit	1:5000
IL-33	R&D	AF3626	Goat	1:2000
Lamin b1	Proteintech	12987-1-AP	Rabbit	1:5000
PENK	Abclonal	A6302	Rabbit	1:2000
P-ERK1/2	Proteintech	80031-1-RR	Rabbit	1:2500
ST2	Abcam	ab25877	Rabbit	1:2000
S100A10	R&D	AF2377	Goat	1:2000
TH	Genetex	GTX113016	Rabbit	1:5000

Table S4. The characteristics of enrolled participants.

	**HC**	**PD**	***P*-value**
Case, n	43	112	
Age, year	56.7 (7.5)	59.1 (9.0)	0.1225
Female (%)	17 (39.5)	59 (52.7)	0.1428
Educational levels, year	9.2 (1.2)	9.6 (1.4)	0.1002
**MMSE**	**27.3 (2.7)**	**23.4 (4.9)**	**<0.0001**
**MoCA**	**24.5 (3.5)**	**19.3 (5.4)**	**<0.0001**
HAMD	11.7 (4.5)	11.8 (5.9)	0.9201
HAMA	11.9 (4.6)	13.2 (7.1)	0.2674
**PSQI**	**5.4 (2.5)**	**7.1 (3.4)**	**0.0033**
WBC, 10^9/L	4.8 (1.6)	5.2 (1.3)	0.1105
Hb, g/L	129.9 (21.5)	130.3 (13.7)	0.8909
hs-CRP, mg/L	0.9 (2.3)	1.4 (3.6)	0.3990
**Glu, mmol/L**	**5.9 (2.0)**	**5.1 (0.7)**	**0.0003**
IL-33, pg/mL	13.6 (5.4)	13.5 (5.7)	0.9211
**sST2, pg/mL**	**4597.6 (1178.1)**	**5702.7 (1632.8)**	**<0.0001**
Disease onset, year	–	55.1 (9.0)	–
Disease duration, year	–	4.0 (3.4)	–
LEDD, mg	–	608.4 (284.4)	–
Midbrain area, cm^2	–	5.4 (0.6)	–
MDS-UPDRS I	–	11.4 (5.9)	–
MDS-UPRDS II	–	17.8 (7.6)	–
MDS-UPDRS III	–	35.0 (12.2)	–

Table S5. The accuracy of plasma sST2 in the diagnosis of PD in the ROC curve.

	Cases	AUC	95%CI	Cut-off	Sens.	Spec.	*P*-value
PD vs. HC	112/43	0.702	0.615~0.788	5441 pg/mL	51.8	79.1	0.0001
PD vs. PSP/MSA	112/17	0.548	0.375~0.710	6911 pg/mL	23.2	97.7	0.5263
PD vs. ET	112/6	0.842	0.664~1.000	3421 pg/mL	93.8	66.7	0.0048
PD vs. Dystonia	112/8	0.708	0.524~0.892	4342 pg/mL	79.5	62.5	0.0497

Table S6. The characteristics of PD patients stratified by H&Y stages.

	H&Y I	H&Y II	H&Y III	*P*-value
Case, n	43	55	14	
Age, year	54.2 (7.8)	61.1 (8.3)	66.4 (7.6)	<0.0001*
Female (%)	21 (48.8)	31 (56.4)	7 (50.0)	0.7429
Educational levels, year	9.7 (1.5)	9.6 (1.4)	9.6 (1.2)	0.9245
Disease onset, year	51.6 (8.2)	56.5 (8.5)	60.3 (9.8)	0.0036*
Disease duration, year	2.6 (2.2)	4.6 (3.8)	6.1 (3.2)	<0.0001*
MDS-UPDRS I	7.6 (3.7)	12.4 (4.0)	19.1 (8.6)	<0.0001*
MDS-UPRDS II	13.1 (6.3)	19.2 (5.3)	26.5 (9.0)	<0.0001*
MDS-UPDRS III	27.3 (11.3)	36.5 (7.9)	52.6 (8.5)	<0.0001*
LEDD, mg	514.0 (257.4)	633.4 (246.1)	800.6 (391.3)	0.0061*
Midbrain area, cm^2	5.4 (0.6)	5.4 (0.5)	5.6 (0.7)	0.5852
MMSE	25.9 (3.4)	23.0 (4.2)	17.1 (5.6)	<0.0001*
MoCA	22.3 (3.1)	18.4 (4.6)	12.0 (5.4)	<0.0001*
HAMD	9.8 (4.2)	12.8 (6.9)	14.2 (4.3)	0.0055*
HAMA	11.1 (6.3)	13.8 (6.8)	17.1 (8.8)	0.0229
PSQI	5.5 (3.3)	7.6 (3.1)	10.1 (2.7)	<0.0001*
IL-33, pg/mL	16.6 (4.6)	12.3 (5.7)	9.3 (3.6)	<0.0001*
sST2, pg/mL	5112.5 (1678.1)	5882.4 (1438.5)	6809.6 (1569.4)	0.0071*
WBC, 10^9/L	5.2 (1.2)	5.2 (1.3)	5.6 (1.6)	0.6730
Neutrophil	2.9 (0.8)	3.0 (1.0)	3.6 (1.7)	0.5599
Lymphocyte	1.8 (0.5)	1.6 (0.4)	1.5 (0.5)	0.2196
NLR	1.7 (0.6)	1.9 (0.7)	3.1 (2.8)	0.1486
Hb, g/L	133.3 (12.3)	127.4 (13.9)	133.8 (15.6)	0.0470
hs-CRP, mg/L	1.0 (1.2)	1.7 (4.9)	1.7 (2.2)	0.5757
Glu，mmol/L	5.0 (0.6)	5.2 (0.8)	5.2 (0.7)	0.1881
Iron metabolism				
Ferritin, μmol/L	201.8 (93.6)	226.0 (184.4)	232.6 (255.9)	0.8049
Serum iron, μg/L	17.3 (5.0)	15.0 (5.1)	13.3 (4.5)	0.0384
Transferrin, g/L	2.2 (0.3)	2.2 (0.3)	2.1 (0.3)	0.9074
sTfR, mg/L	2.9 (0.9)	3.0 (1.0)	3.2 (0.9)	0.4281
CER, g/L	0.26 (0.05)	0.26 (0.06)	0.29 (0.10)	0.6534

*The statistical differences still existed significantly after Bonferroni correction ($P < 0.0167$).

Table S7. The association of plasma IL-33 or sST2 levels with PD features.

	IL-33*		sST2*	
	**β**	***P*-value**	**β**	***P*-value**
MDS-UPDRS I	-0.247	0.0278	56.96	0.0342
MDS-UPRDS II	-0.183	0.0223	50.85	0.0172
MDS-UPDRS III	-0.162	0.0017	34.64	0.0061
LEDD	-0.001	0.7570	1.30	0.0140
Midbrain area	-0.407	0.7178	-789.15	0.0039
MMSE	0.124	0.9213	-83.39	0.0114
MoCA	0.164	0.1538	-68.94	0.0242
HAMD	0.010	0.9176	30.35	0.2398
HAMA	-0.015	0.8580	18.90	0.3820
PSQI	-0.528	0.0032	81.43	0.0743
WBC	0.300	0.5081	346.09	0.0034
Neutrophil	0.582	0.2971	494.23	0.0008
Lymphocyte	-0.273	0.8276	156.44	0.6334
NLR	0.325	0.5490	385.49	0.0078
Hb	-0.002	0.9717	15.12	0.3121
hs-CRP	0.021	0.9050	102.13	0.0234
Glu	0.171	0.8794	93.66	0.6710
Iron metabolism				
Ferritin	0.002	0.6620	0.45	0.7018
Serum iron	0.102	0.4437	-49.56	0.1711
Transferrin	0.929	0.6773	-1515.09	0.0148
sTfR	-0.209	0.7610	-71.25	0.7000
CER	4.011	0.7236	-481.27	0.8764

*The models were adjusted for age, gender, educational levels, years of PD onset, disease duration, medical history, smoking, and alcohol consumption.

Table S8. The sample characteristics of astrocytes in the RNA seq.

Group	Cases	Sample	Clean reads	**GC percent**
SiNC_PBS	4	SiNC_PBS-1	44236350	52.05
		SiNC_PBS-2	45280360	52.18
		SiNC_PBS-3	44430138	52.12
		SiNC_PBS-4	44861138	51.90
SiNC_MPP^+	4	SiNC_MPP^+-1	42565144	51.14
		SiNC_MPP^+-2	43429906	50.81
		SiNC_MPP^+-3	45013522	51.98
		SiNC_MPP^+-4	44981330	50.88
Si*Il33*_PBS	3	Si*Il33*_PBS-1	45029516	52.58
		Si*Il33*_PBS-2	44954628	52.29
		Si*Il33*_PBS-3	45736508	51.89
Si*Il33*_ MPP^+	3	Si*Il33*_ MPP^+-1	43183746	50.59
		Si*Il33*_ MPP^+-2	42511664	50.26
		Si*Il33*_ MPP^+-3	40321010	50.44

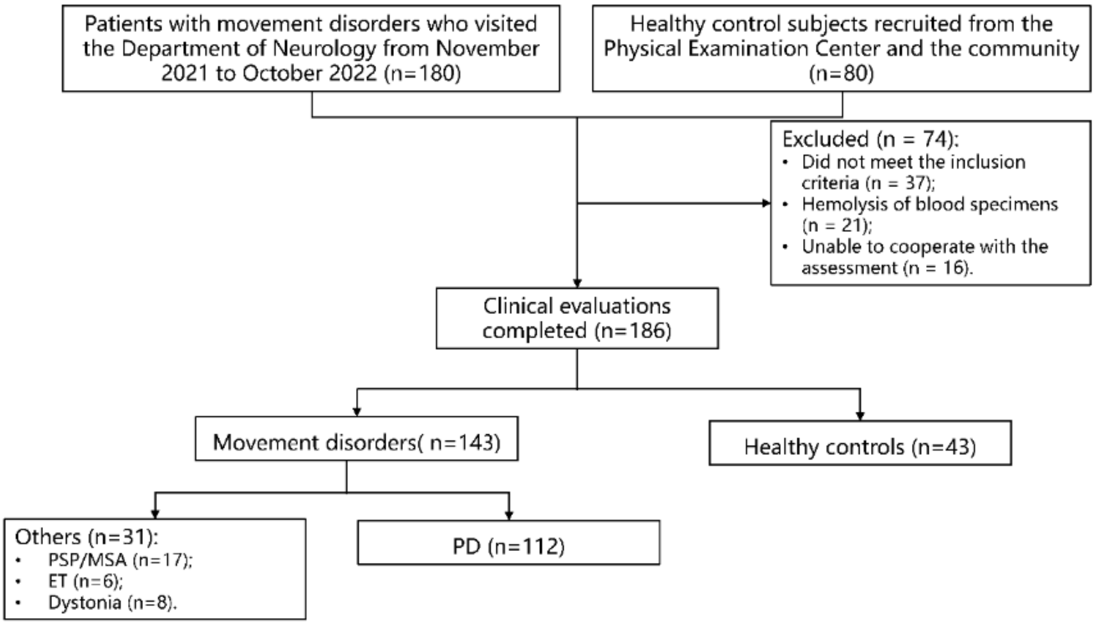

Fig. S1. Flowchart of included participants.

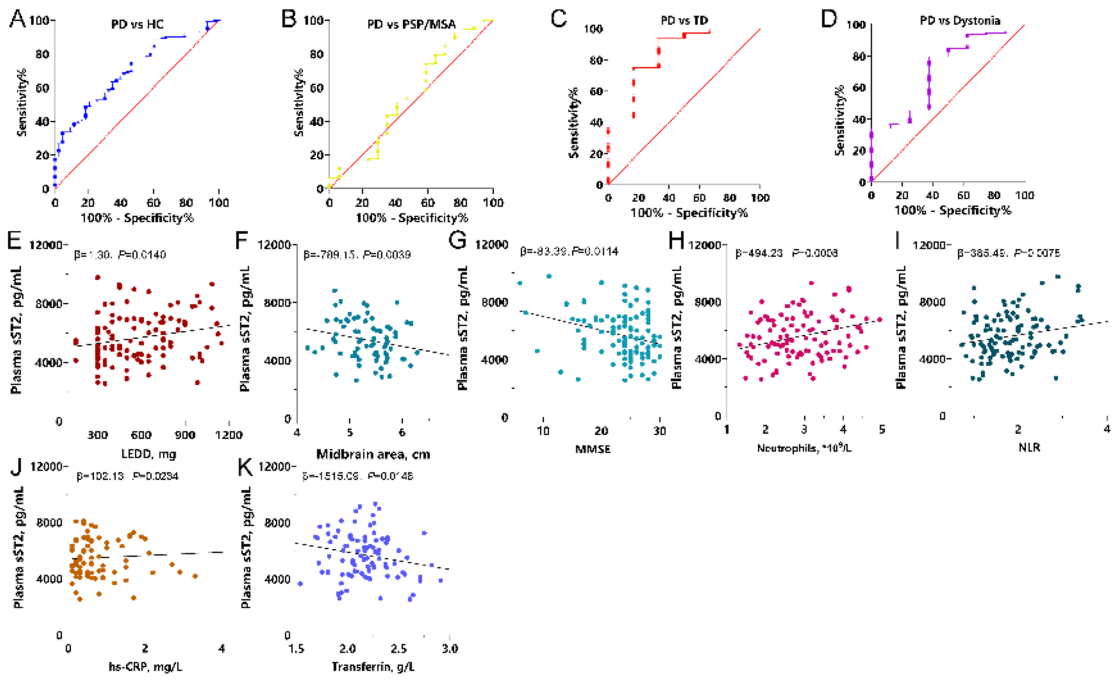

Fig. S2. Associations of plasma IL-33 and sST2 levels with PD symptoms. A-D. High discriminatory power was found in distinguishing PD from HC (AUC = 0.702; B), ET (AUC = 0.708; D), and dystonia (AUC = 0.842; E), while inferior power in differentiating from PSP/MSA (AUC = 0.548; C); **E-K.** Higher levels of sST2 were associated with more LEDD intake (*P* = 0.0140, E), lower midbrain area (*P* = 0.0039; F), worsen MMSE score (*P* = 0.0114; G), higher neutrophils count (*P* = 0.0008; H), NLR (*P* = 0.0078; I), hs-CRP (*P* = 0.0234; J) and lower Transferrin (*P* = 0.0148; K).

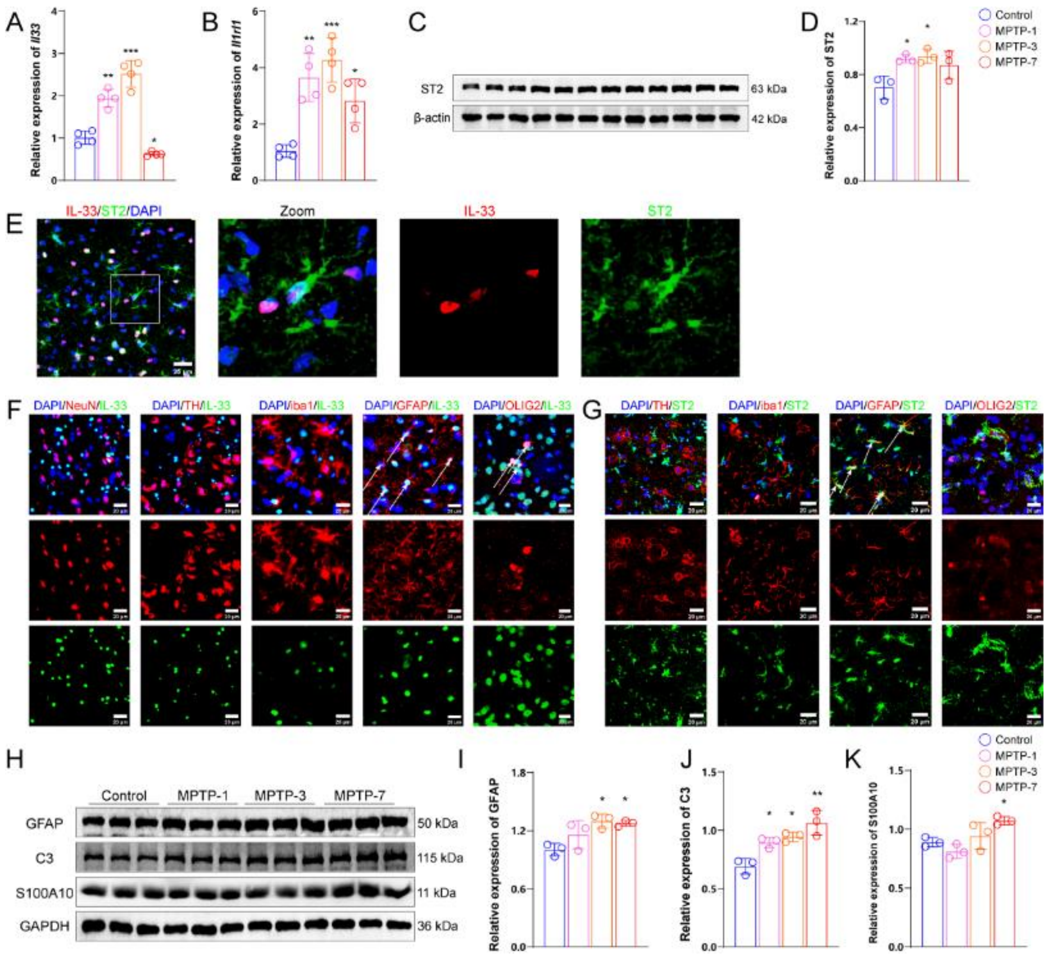

Fig. S3. The expression of IL-33 and ST2 in the substantia nigra. A. qPCR analyses of mRNA expression of *Il33* in the SN, $n = 4$ per group; **B.** qPCR analyses of mRNA expression of *Il1rl1* in the SN, $n = 4$ per group; **C, D.** Protein expression of ST2 in the SN assessed by WB (C) and quantitative analyses (D) among groups, $n = 3$ per group; **E.** IF stanning of IL-33 with ST2, scale bar = 25 μm; **F.** Double immunofluorescence staining of IL-33 with NeuN (neuron marker), TH (DA neuron marker), Iba1 (microglia marker), GFAP (astrocyte marker), and Olig2 (oligodendrocyte marker), scale bar = 20 μm; **G.** Double immunofluorescence staining of ST2 with TH (DA neuron marker), Iba1 (microglia marker), GFAP (astrocyte marker), and Olig2 (oligodendrocyte marker), scale bar = 20 μm; **H-K.** Protein expression of GFAP, C3, and S100A10 in the SN assessed by WB (H) and quantitative analyses (I-K) among groups, $n = 3$ per group; I. Representative IF images of IL-33 with GFAP in the SN, scale bar = 25 μm; Data are presented as mean ± SD. Significances were assessed using two-way ANOVA, $^{*}P < 0.05$, $^{**}P < 0.01$, $^{***}P < 0.001$, $^{****}P < 0.0001$.

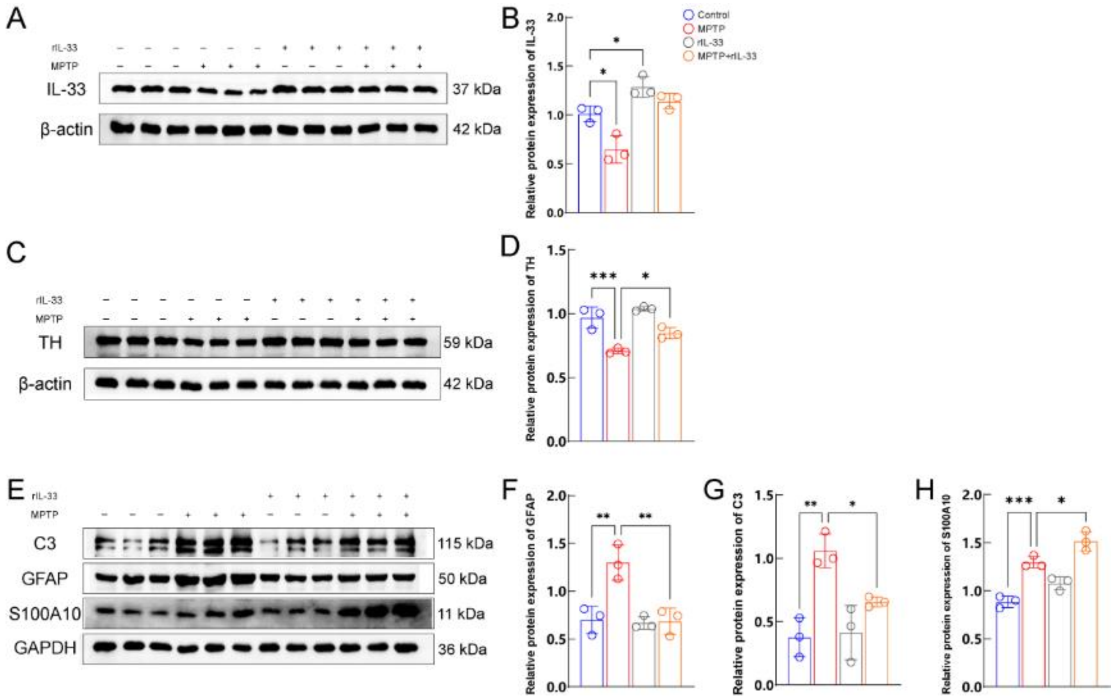

Fig. S4. rIL-33 supplementation ameliorates DA neuron loss and neuroinflammation in the MPTP mouse model. A, B. Protein expression of IL-33 in the SN after rIL-33 administration assessed by WB (A) and quantitative analyses (B) among groups; **C, D.** Protein expression of TH in the SN assessed by WB (H) and quantitative analyses (I) among groups; **E-H.** Protein expression of GFAP, C3, and S100A10 in the SN assessed by WB (N) and quantitative analyses (O-Q) among groups; *n* = 3 per group. Data are presented as mean ± SD; Significances were assessed using two-way ANOVA, **P* < 0.05, ***P* < 0.01, ****P* < 0.001, *****P* < 0.0001.

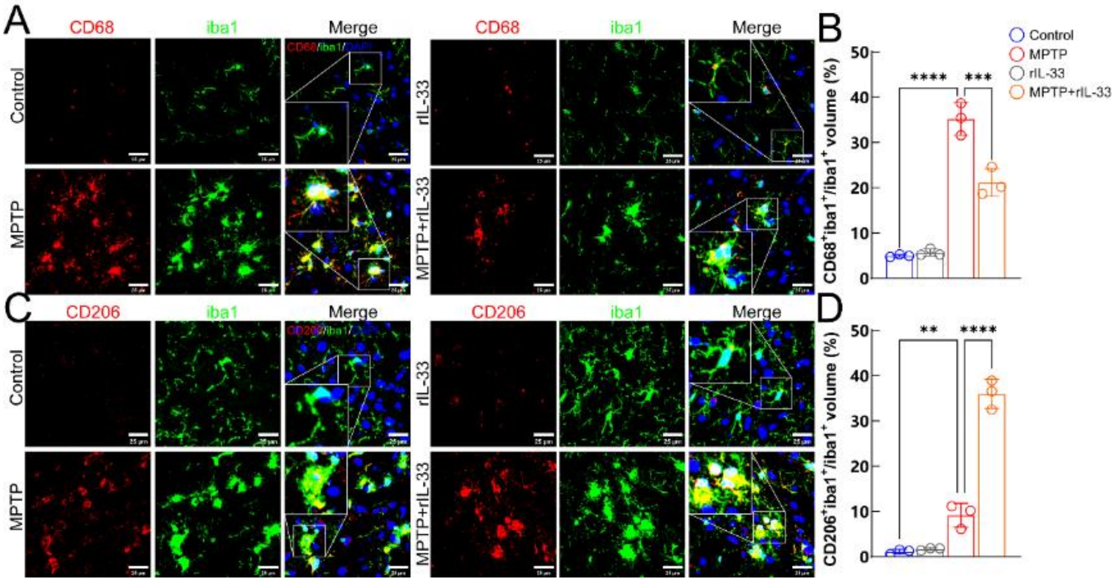

Fig. S5. rIL-33 supplementation improves microglial neuroinflammation in MPTP mouse model. A. Representative confocal images of CD68 (red) and iba1 (green) in the SN, scale bar = 25 μm; **B.** Quantitative analysis of the ratio of CD68$^+$iba1$^+$ to iba1$^+$ area in the SN, $n = 3$ per group; **C.** Representative confocal images of CD206 (red) and iba1 (green) in the SN, scale bar = 25 μm; **D.** Quantitative analysis of the ratio of CD206$^+$iba1$^+$ to iba1$^+$ area in the SN, $n = 3$ per group; Significances were assessed using two-way ANOVA. *$P < 0.05$, **$P < 0.01$, ***$P < 0.001$, ****$P < 0.0001$.

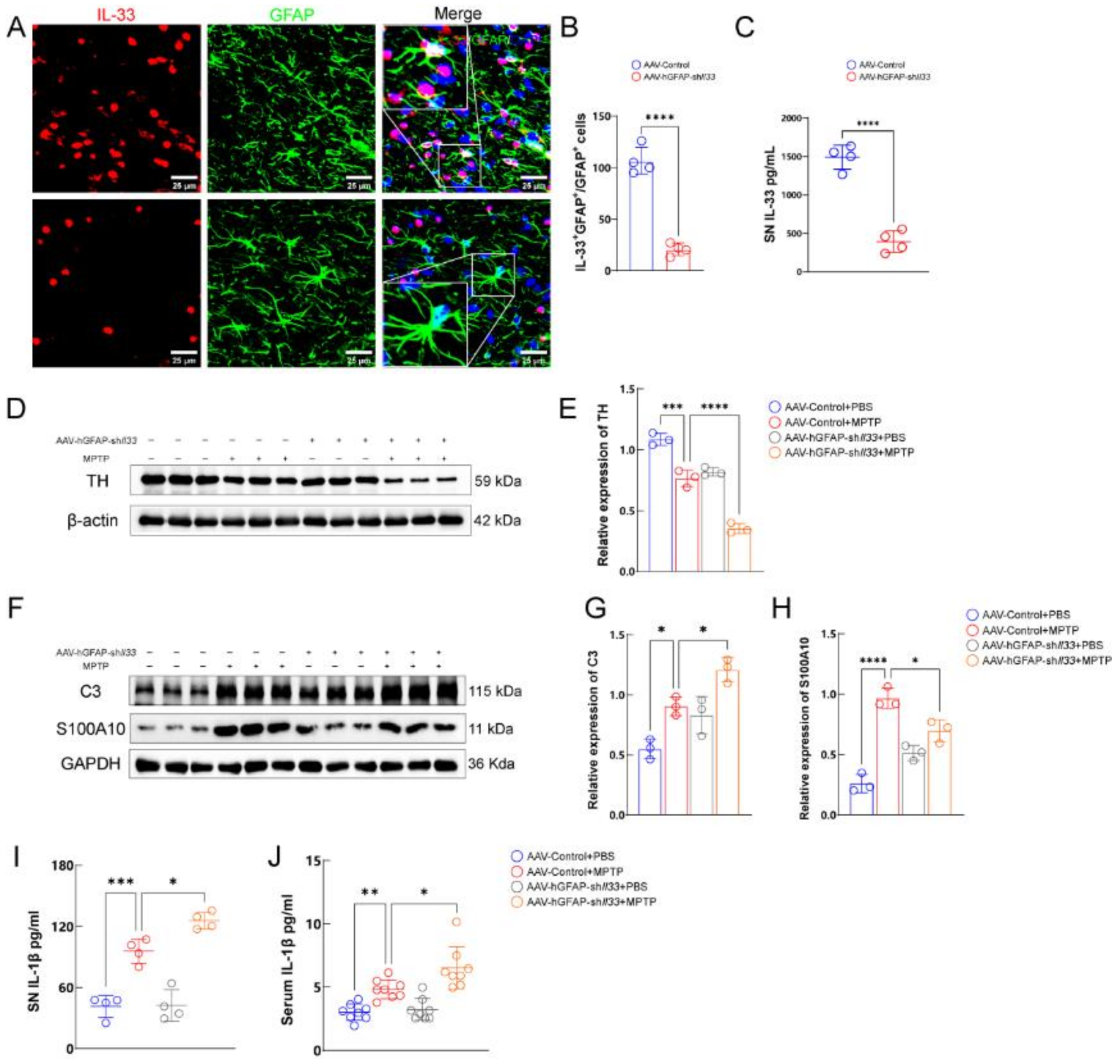

Fig. S6. Astrocyte-specific *Il33* knockdown aggravates PD-like symptoms and neuroinflammation in MPTP mouse model. A. Representative IF images of IL-33 with GFAP in the SN, scale bar = 25 μm; **B.** Quantification of IL-33 average intensity in groups, $n = 4$ per group; **C.** Quantification of IL-33 levels in the SN detected by ELISA, $n = 4$ per group; **D, E.** Protein expression of TH in the SN assessed by WB (D) and quantitative analyses (E) among groups, $n = 3$ per group; **F-H.** Protein expression of C3 and S100A10 in the SN assessed by WB (F) and quantitative analyses (G, H) among groups, $n = 3$ per group; **I.** Quantification of IL-1β levels in the SN detected by ELISA, $n = 4$ per group; **J.** Quantification of IL-1β levels in the serum detected by ELISA, $n = 8$ per group; Data are presented as mean ± SD; Significances were assessed using two-way ANOVA. *$P < 0.05$, **$P < 0.01$, ***$P < 0.001$, ****$P < 0.0001$.

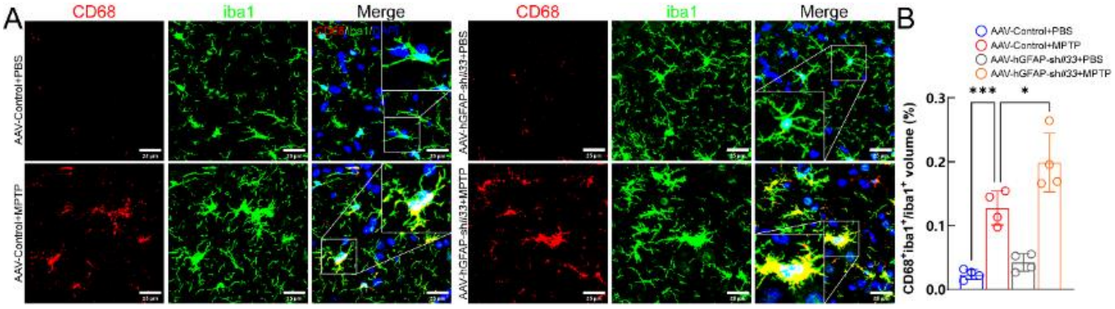

Fig. S7. Astrocyte-specific *Il33* knockdown aggravates microglial neuroinflammation in MPTP mouse model. A. Representative confocal images of CD68 (red) and iba1 (green) in the SN, scale bar = 25 μm; **B.** Quantitative analysis of the ratio of $CD68^{+}iba1^{+}$ to $iba1^{+}$ area in the SN, $n = 3$ per group; Significances were assessed using two-way ANOVA. $*P < 0.05$, $**P < 0.01$, $***P < 0.001$, $****P < 0.0001$.

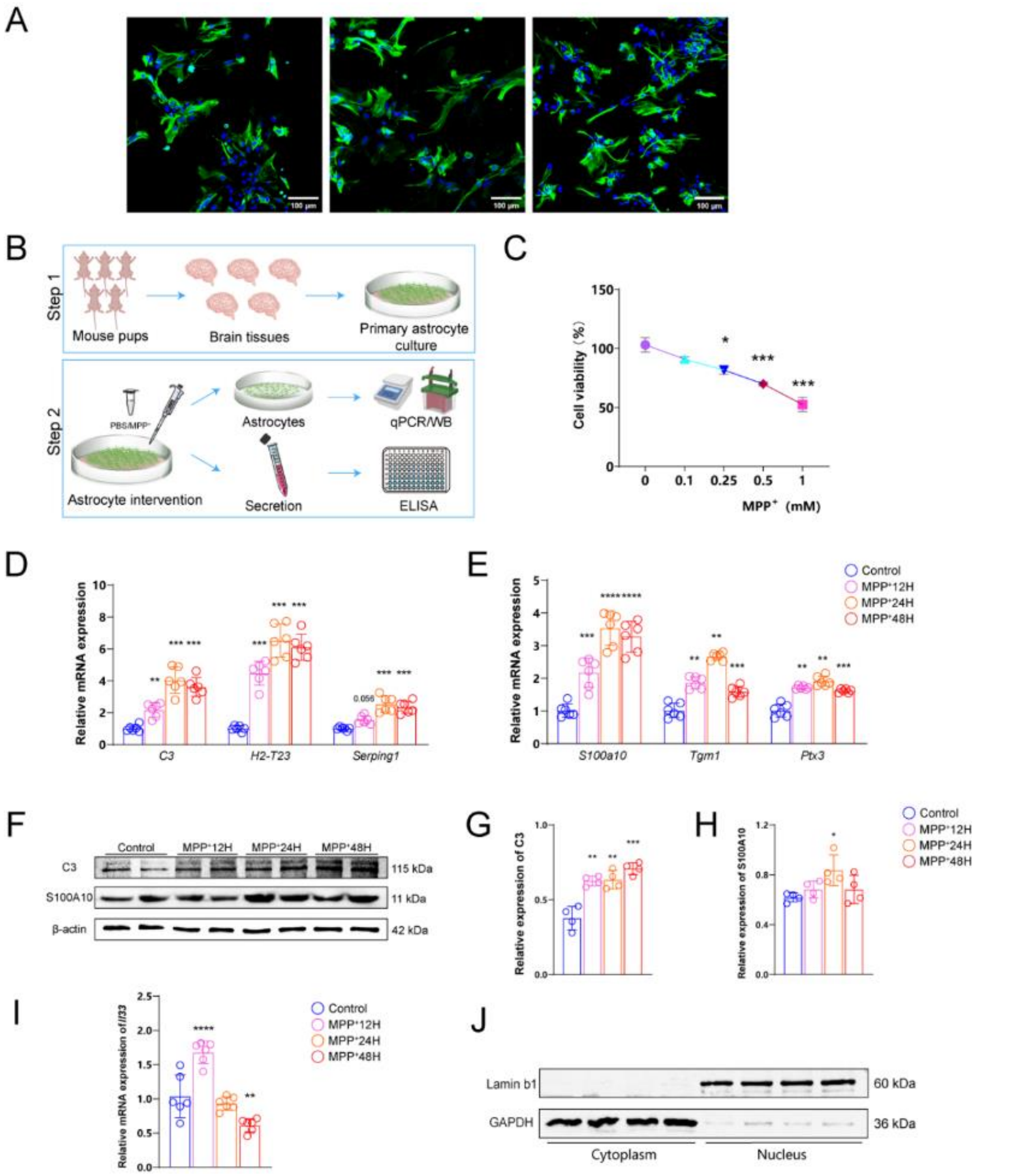

Fig. S8. Dynamic expression of A1/A2 markers and IL-33 in astrocytes. A. Representative IF images of GFAP to detect the purity in primary cultured astrocytes, scale bar = 100 μm; **B.** Flowchart illustrating the construction of MPP$^+$-stimulated astrocyte model; **C.** Cell viability of MPP$^+$-treated astrocytes in different concentrations assessed by CCK-8; **D.** qPCR analyses of mRNA expression of *C3*, *H2-T23* and *Serping1*, $n = 6$ per group; **E.** qPCR analyses of mRNA expression of *S100a10*, *Tgm1* and *Ptx3*, $n = 6$ per group; **F-H.** Protein expression of C3 and S100A10 in astrocytes was assessed by WB (F) and quantitative analyses (G, H) among groups, $n = 4$ per group; I. qPCR analyses of mRNA expression of *Il33*, $n = 4$ per group; **J.** WB results showed the reference protein expression in the nuclear and cytoplasm. *$P < 0.05$, **$P < 0.01$, ***$P < 0.001$, ****$P < 0.0001$.

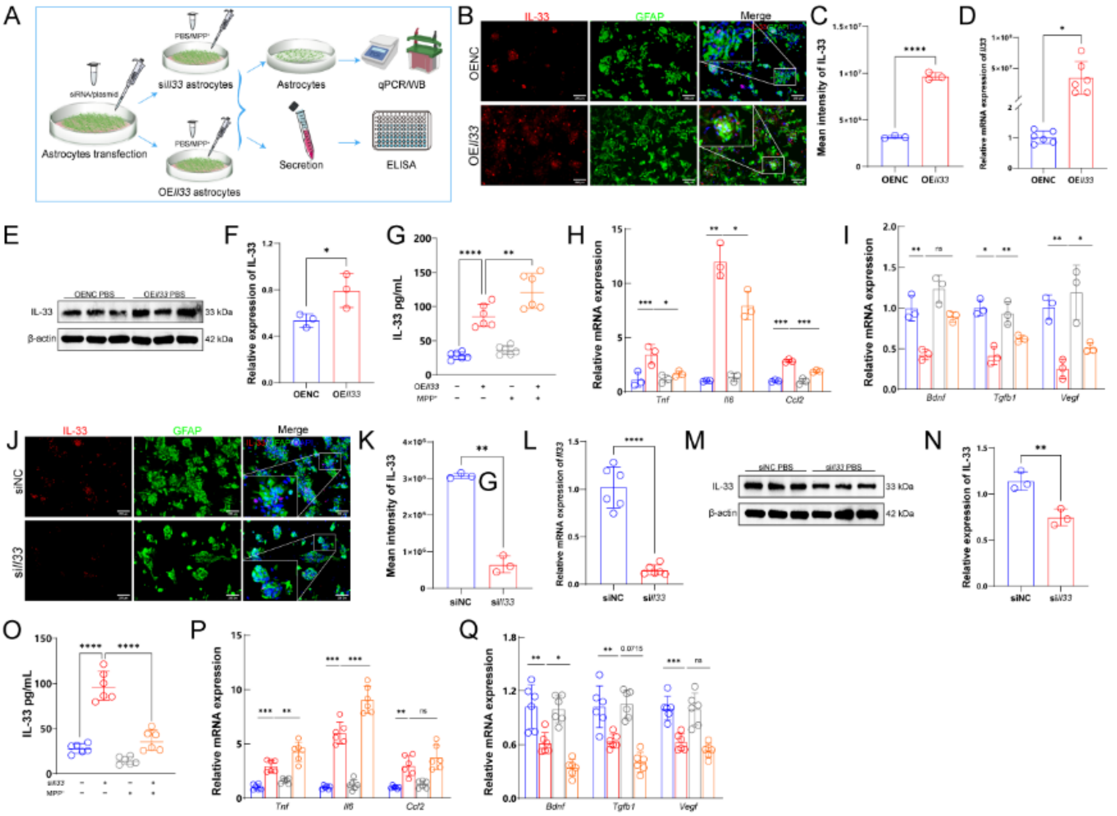

Fig. S9. The validation of the astrocytes model with *Il33* expression and the phenotypic transformation of astrocytes. A. Flowchart illustrating the construction of MPP^+-stimulated astrocyte model with *Il33* gene interference; **B.** Representative IF images of IL-33 (red) with GFAP (green) in OE*Il33* astrocytes scale bar = 100 μm; **C.** Quantification of IL-33 average intensity in groups, *n* = 3 per group; **D.** qPCR analyses of mRNA expression of *Il33* in OE*Il33* astrocytes, *n* = 6 per group; **E, F.** Protein expression of IL-33 in OE*Il33* astrocytes assessed by WB (E) and quantitative analyses (F) among groups, *n* = 3 per group; **G.** Quantification of IL-33 levels after *Il33* overexpression in astrocyte-secreted supernatant detected by ELISA, *n* = 6 per group; **H.** qPCR analyses of mRNA expression of *Tnf*, *Il6* and *Ccl2*, *n* = 4 per group; **I.** qPCR analyses of mRNA expression of *Bdnf*, *Tgfb1* and *Vegf*, *n* = 4 per group; **J.** Representative IF images of IL-33 (red) with GFAP (green) in si*Il33* astrocytes, scale bar = 100 μm; **K.** Quantification of IL-33 average intensity in groups, *n* = 3 per group; **L.** qPCR analyses of mRNA expression of *Il33* in si*Il33* astrocytes, *n* = 6 per group; **M, N.** Protein expression of IL-33 in si*Il33* astrocytes assessed by WB (M) and quantitative analyses (N) among groups, *n* = 3 per group; **O.** Quantification of IL-33 levels after *Il33* knockdown in astrocyte-secreted supernatant detected by ELISA, *n* = 6 per group; **P.** qPCR analyses of mRNA expression of *Tnf*, *Il6* and *Ccl2*, *n* = 4 per group; **Q.** qPCR analyses of mRNA

expression of *Bdnf*, *Tgfb1* and *Vegf*, $n = 4$ per group; Data are presented as mean ± SD; Significances were assessed using two-way ANOVA. *$P < 0.05$, **$P < 0.01$, ***$P < 0.001$, ****$P < 0.0001$.

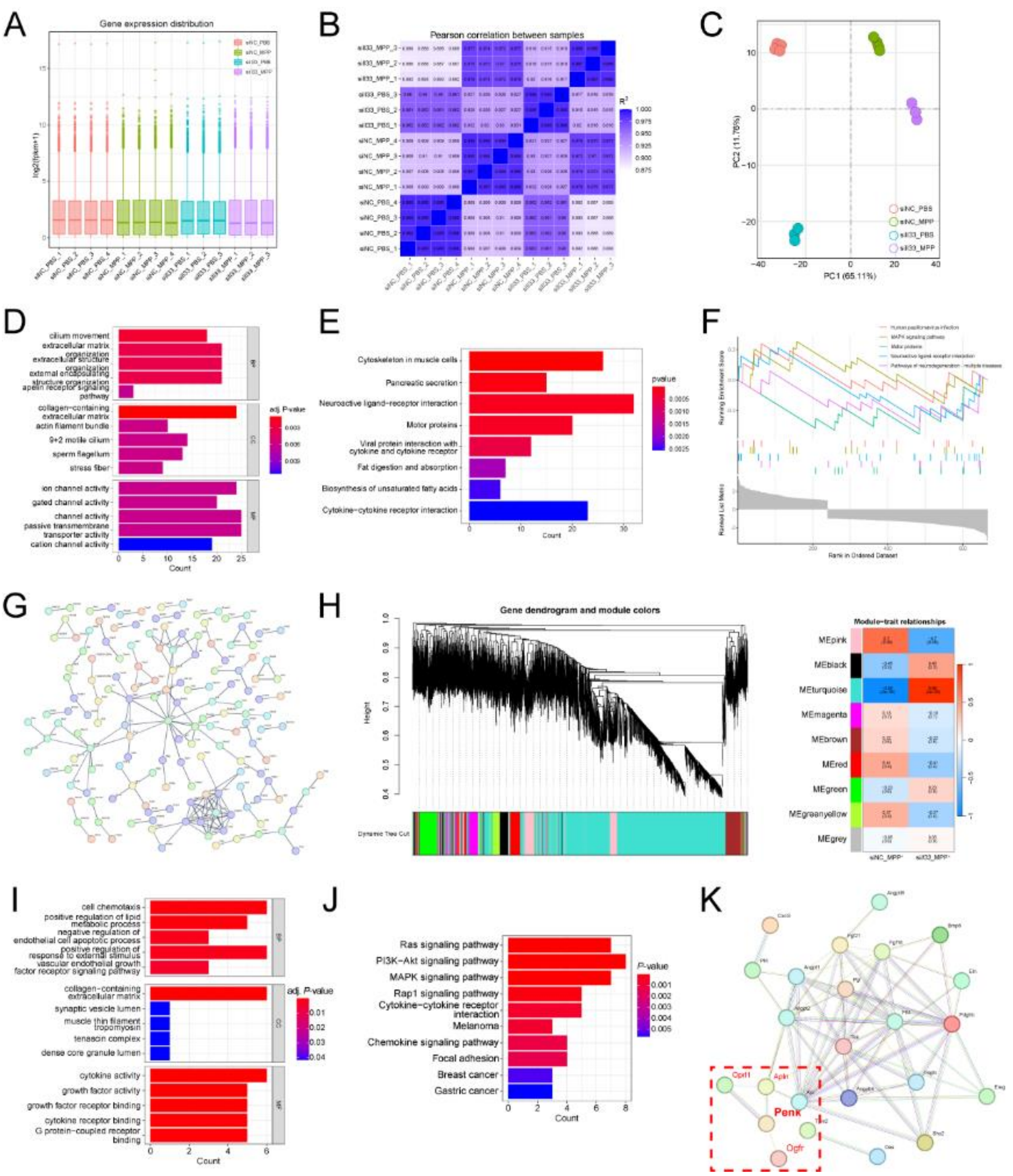

Fig. S10. Identification of key gene in si*Il33* astrocytes. A. Violin plot with median markers depicting gene expression distributions across samples; **B.** Pearson correlation heatmap of normalized expression profiles (Z-score transformed); **C.** PCA analysis plot demonstrating the separation among groups; **D.** Top 5 biological processes, cellular component and molecular function of GO terms in DEGs; **E.** KEGG pathway with DEGs; **F.** GSEA enrichment analysis of DEGs; **G.** PPI analysis of DEGs; **H.** WGCNA network and module detection; **I.** Top 5 biological processes, cellular components, and molecular functions of GO terms in hub genes; **J.** KEGG pathway with hub genes; **K.** PPI analysis of hub genes.

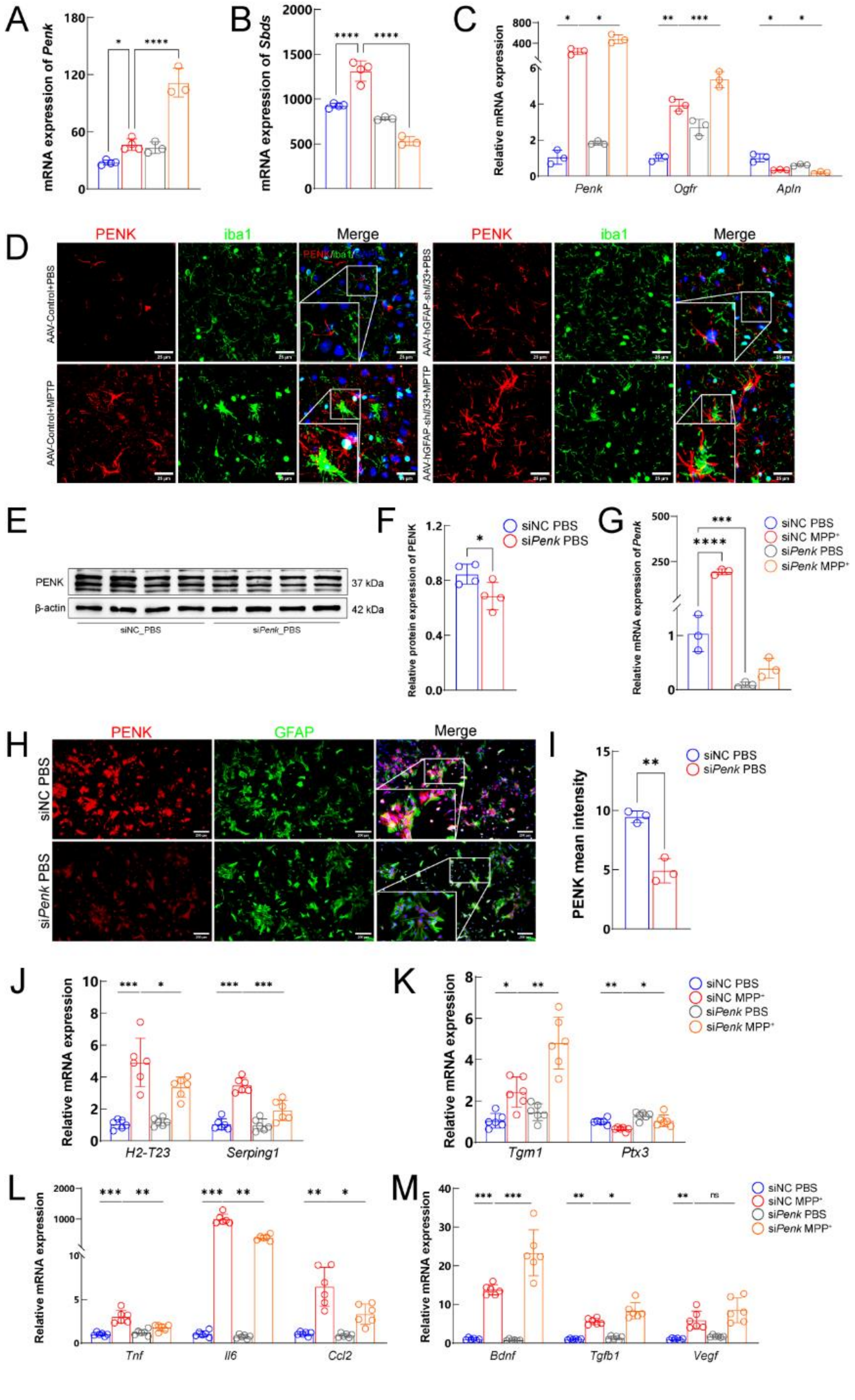

A
mRNA expression of Penk
180
120
60
0
*
****
B
mRNA expression of Sbds
2000
1500
1000
500
0
****
****
C
Relative mRNA expression
800
400
6
4
2
0
Penk
Ogfr
Apln
D
PENK
iba1
Merge
AAV-Control+PBS
AAV-Control+MPTP
AAV-hGFAP-shIl33+PBS
AAV-hGFAP-shIl33+MPTP
E
PENK
β-actin
37 kDa
42 kDa
siNC_PBS
siPenk_PBS
F
Relative protein expression of PENK
1.2
0.8
0.4
0.0
siNC PBS
siPenk PBS
G
Relative mRNA expression of Penk
500
250
1
0
***
****
siNC PBS
siNC MPP+
siPenk PBS
siPenk MPP+
H
PENK
GFAP
Merge
siNC PBS
siPenk PBS
I
PENK mean intensity
15
10
5
0
**
siNC PBS
siPenk PBS
J
Relative mRNA expression
10
8
6
4
2
0
H2-T23
Serping1
K
Relative mRNA expression
8
6
4
2
0
Tgm1
Ptx3
siNC PBS
siNC MPP+
siPenk PBS
siPenk MPP+
L
Relative mRNA expression
2000
1000
10
5
0
Tnf
Il6
Ccl2
M
Relative mRNA expression
40
30
20
10
0
Bdnf
Tgfb1
Vegf
ns
siNC PBS
siNC MPP+
siPenk PBS
siPenk MPP+

Fig. S11. The expression and role of PENK in astrocytes. A. RNA-seq mRNA expression of *Penk*, *n* = 3 per group; **B.** RNA-seq mRNA expression of *Sbds*, *n* = 3 per group; **C.** qPCR analyses of mRNA expression of *Penk*, *Ogfr* and *Apln* in si*Il33* astrocytes, *n* = 4 per group; **D.** Representative confocal images of PENK (red) and Iba1 (green) in the SN, scale bar = 25 μm; **E, F.** Protein expression of PENK in si*Penk* astrocytes assessed by WB (E) and quantitative analyses (F) among groups, *n* = 3 per group; **G.** qPCR analyses of mRNA expression of *Penk* in si*Penk* astrocytes, *n* = 3 per group; **H.** Representative IF images of PENK (red) with GFAP (green) in si*Penk* astrocytes scale bar = 100 μm; **I.** Quantification of PENK average intensity in groups, *n* = 3 per group; **J.** qPCR analyses of mRNA expression of *H2T23* and *Serping1*, *n* = 6 per group; **K.** qPCR analyses of mRNA expression of *Tgm1* and *Ptx3*, *n* = 6 per group; **L.** qPCR analyses of mRNA expression of *Tnf*, *Il6* and *Ccl2*, *n* = 6 per group; **M.** qPCR analyses of mRNA expression of *Bdnf*, *Tgfb1* and *Vegf*, *n* = 6 per group; Data are presented as mean ± SD; Significances were assessed using two-way ANOVA. *$P < 0.05$, **$P < 0.01$, ***$P < 0.001$, ****$P < 0.0001$.

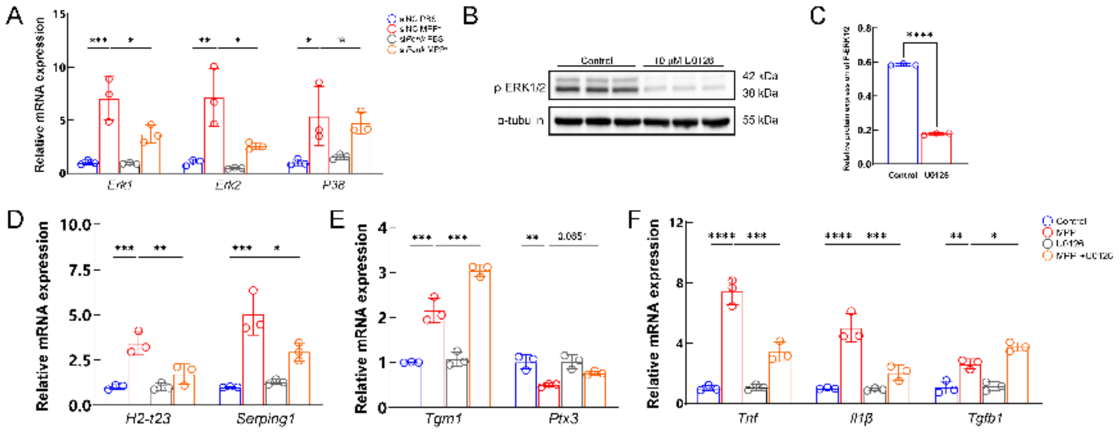

Fig. S12. The role of PENK-ERK/MAPK pathway in astrocytes. A. qPCR analyses of mRNA expression of *Erk1*, *Erk2* and *P38*, *n* = 3 per group; **B, C.** Protein expression of p-ERK1/2 in astrocytes assessed by WB (B) and quantitative analyses (C) among groups, *n* = 3 per group; **D.** qPCR analyses of mRNA expression of *H2T23* and *Serping1*, *n* = 3 per group; **E.** qPCR analyses of mRNA expression of *Tgm1* and *Ptx3*, *n* = 3 per group; F. qPCR analyses of mRNA expression of *Tnf*, *Il1β* and *Tgfb1*, *n* = 3 per group; Data are presented as mean ± SD; Significances were assessed using two-way ANOVA. **P* < 0.05, ***P* < 0.01, ****P* < 0.001, *****P* < 0.0001.
